# Smartphone-Based Interventions for Physical Activity Promotion: Scoping Review of the Evidence Over the Last 10 Years

**DOI:** 10.2196/24308

**Published:** 2021-07-21

**Authors:** Alex Domin, Donna Spruijt-Metz, Daniel Theisen, Yacine Ouzzahra, Claus Vögele

**Affiliations:** 1 Research Group: Self-Regulation and Health Department of Behavioural and Cognitive Sciences University of Luxembourg Esch-sur-Alzette Luxembourg; 2 USC mHealth Collaboratory Center for Economic and Social Research University of Southern California Los Angeles, CA United States; 3 ALAN – Maladies Rares Luxembourg Kockelscheuer Luxembourg; 4 Research Support Department University of Luxembourg Esch-sur-Alzette Luxembourg

**Keywords:** scoping review, smartphone application, physical activity, behavior change, mobile health, research design, mHealth, adolescents, adults, BCT, mobile phonescoping review, smartphone application, physical activity, behavior change, mobile health, research design, mHealth, adolescents, adults, BCT, mobile phone

## Abstract

**Background:**

Several reviews of mobile health (mHealth) physical activity (PA) interventions suggest their beneficial effects on behavior change in adolescents and adults. Owing to the ubiquitous presence of smartphones, their use in mHealth PA interventions seems obvious; nevertheless, there are gaps in the literature on the evaluation reporting processes and best practices of such interventions.

**Objective:**

The primary objective of this review is to analyze the development and evaluation trajectory of smartphone-based mHealth PA interventions and to review systematic theory- and evidence-based practices and methods that are implemented along this trajectory. The secondary objective is to identify the range of evidence (both quantitative and qualitative) available on smartphone-based mHealth PA interventions to provide a comprehensive tabular and narrative review of the available literature in terms of its nature, features, and volume.

**Methods:**

We conducted a scoping review of qualitative and quantitative studies examining smartphone-based PA interventions published between 2008 and 2018. In line with scoping review guidelines, studies were not rejected based on their research design or quality. This review, therefore, includes experimental and descriptive studies, as well as reviews addressing smartphone-based mHealth interventions aimed at promoting PA in all age groups (with a subanalysis conducted for adolescents). Two groups of studies were additionally included: reviews or content analyses of PA trackers and meta-analyses exploring behavior change techniques and their efficacy.

**Results:**

Included articles (N=148) were categorized into 10 groups: commercial smartphone app content analyses, smartphone-based intervention review studies, activity tracker content analyses, activity tracker review studies, meta-analyses of PA intervention studies, smartphone-based intervention studies, qualitative formative studies, app development descriptive studies, qualitative follow-up studies, and other related articles. Only 24 articles targeted children or adolescents (age range: 5-19 years). There is no agreed evaluation framework or taxonomy to code or report smartphone-based PA interventions. Researchers did not state the coding method, used various evaluation frameworks, or used different versions of behavior change technique taxonomies. In addition, there is no consensus on the best behavior change theory or model that should be used in smartphone-based interventions for PA promotion. Commonly reported systematic practices and methods have been successfully identified. They include PA recommendations, trial designs (randomized controlled trials, experimental trials, and rapid design trials), mixed methods data collection (surveys, questionnaires, interviews, and focus group discussions), scales to assess app quality, and industry-recognized reporting guidelines.

**Conclusions:**

Smartphone-based mHealth interventions aimed at promoting PA showed promising results for behavior change. Although there is a plethora of published studies on the adult target group, the number of studies and consequently the evidence base for adolescents is limited. Overall, the efficacy of smartphone-based mHealth PA interventions can be considerably improved through a more systematic approach of developing, reporting, and coding of the interventions.

## Introduction

### Background

Physical inactivity has been identified as a *global pandemic* and is reported to be the fourth leading cause of death worldwide [1]. There is strong evidence that physical inactivity shortens life expectancy and increases the risk of noncommunicable diseases such as breast and colon cancers, type 2 diabetes, and coronary heart disease, resulting in 5.3 million deaths annually worldwide [2]. Moreover, the world economy suffers great financial losses because of physical inactivity, bearing a yearly estimated burden of US $53.8 billion health care costs worldwide [3]. To avoid these health and financial consequences, it is important to pursue pre-emptive strategies to identify and mitigate the causes of low levels of physical activity (PA).

At the same time, the world is facing another life-threatening pandemic caused by SARS‑CoV‑2 or COVID‑19 [4]. The World Health Organization (WHO) declared the virus outbreak as a pandemic on March 11, 2020, and more than 5.5 million cases of COVID-19 worldwide have been reported since, resulting in more than 346,600 deaths as of May 26, 2020 [5]. As a response to this crisis, many governments introduced confinements, curfews, or quarantines as compulsory or recommended containment and prevention measures [6]. Several studies have since found that home quarantine introduces a shift in lifestyle toward limited socialization and reduced PA, which may contribute to an exacerbation of already reduced PA levels in the population and its associated health risks [7,8].

Although confinement measures have been introduced to reduce the spread of the virus, with some success in flattening the curve, these interventions to contain the COVID-19 outbreak have unsurprisingly resulted in an increased use of digital communication technologies, such as in mobile health (mHealth) and telehealth approaches in the domains of PA and medicine [9-12]. In light of these developments, and the resulting increase in the importance of digital technologies for health, it has become even more evident that it is crucial to significantly advance the field of mHealth PA technologies by identifying knowledge gaps, evaluating reporting processes, and establishing best practices. This scoping review, therefore, focuses on the analysis of the development and evaluation trajectory of mHealth PA interventions and on the review of systematic theory- and evidence-based practices and methods that are implemented along this trajectory. We describe the advantages and disadvantages of theory- and evidence-based practices and methods to present recommendations on how to improve and accelerate the overall process of the development and evaluation of mHealth PA interventions. The overall aim of this review is to provide guidance in the field of smartphone- and wearable-based mHealth PA interventions.

A major decline in PA levels occurs during the transition from childhood to adolescence [13,14]. A high percentage of the global population of adolescents does not reach the levels of PA recommended by the WHO [15,16]. Insufficient levels of PA tend to track through childhood and adolescence into adulthood [17-19]. According to the report *Health at a Glance: Europe 2016* from the Organization for Economic Co-operation and Development, 36% of the adult population of the European Union does not meet the recommended levels of PA. According to the same report, the majority of reported adolescents in the European Union, by the age of 15, do not even reach 30% of the recommended PA time [16]. Given the scale of the problem and the fact that higher PA is associated with physical [20] and mental [21] health benefits, it is important to develop interventions that can effectively support and promote PA, which can reach large numbers of people easily and that can do this low-touch or remotely, and at low cost.

Face-to-face interventions are resource intensive and limited because of their attachment to their specific environment and multicomponent nature [22]. They can be difficult to access depending on circumstances such as a busy schedule, illness, childcare, lack of safe and attractive spaces to exercise, or, as has now been demonstrated, disasters such as the COVID-19 pandemic. Smartphones and affordable wearable sensors have become ubiquitous in the lives of today’s population [23]. These devices could be beneficial for the development and delivery of remote PA interventions [22,24,25]. The advantages of smartphones and devices integrated into smartphone platforms include the ability to schedule the delivery of intervention content that can take into account the time of day and momentary environment of the user. These technologies offer the possibility of high-level personalization toward the user and the unobtrusive and in situ collection of behavioral data [26]. Therefore, smartphone-based interventions are accessible, scalable, comparatively inexpensive, and can deliver low-touch or completely remote interventions. These features make smartphone-based interventions more advantageous for self-monitoring of PA compared with stand-alone pedometers and are preferable over computer-based interventions [27,28]. Several reviews of smartphone-based interventions have outlined their acceptability, efficacy, and effectiveness in increasing health behaviors in several age groups [24,29,30].

Despite their strong potential, the evidence concerning smartphone-based interventions to improve PA and decrease sedentary behaviors (SBs) is only emerging, and the literature is poorly systematized, which results in methodological inconsistencies and significant gaps in our understanding of the developments in the field of mHealth PA interventions.

### Prior Work

There are four recent scoping reviews, which attempted to address these gaps [31-34]. Lee et al [34] aimed to identify the *efficacy* and *effectiveness* of mHealth PA interventions in adolescents; Aromatario et al [33] investigated how researchers conducting studies with mHealth PA and diet apps as a main component assess the app *conditions of* *effectiveness* across age groups; McCallum et al [32] explored the extent to which evaluations of mHealth PA apps and wearables affect the *effectiveness*, *engagement*, *and acceptability* of these apps, and Ly [31] reviewed the literature with the aim of presenting *an account of the current knowledge* on the use of mHealth interventions to enhance PA levels in young adults. These reviews included studies evaluating a range of different target populations with various states of health or ill health (without illness; chronic illness [33], including attention deficit/hyperactivity disorder [34]; cancer and diabetes [32]; and acute illness [33]) while targeting either PA alone [31], diet alone [32], or a combination of the two [33]. Finally, yet importantly, almost all reviews (excluding Aromatario et al [33], who focused on mHealth app only) included studies with various modes of delivery of the intervention, such as smartphone apps, websites, SMS text message, tablets, and PDAs.

Although these reviews are informative and have their strengths in different areas, they still fail to provide answers to several questions. First, behavior change components of mHealth interventions are often conceptualized as behavior change techniques (BCTs), which are described systematically in various BCT taxonomies [35-37]. However, there is no consensus on a universally accepted behavior change taxonomy. Therefore, it remains unclear why certain authors prefer one taxonomy to another. Second, studies on smartphone-based interventions fall under the domain of mHealth, which is commonly defined as a *medical and public health practice supported by mobile devices, such as mobile phones, patient monitoring devices, PDAs, and other wireless* *devices* [38]. This definition is currently argued to be outdated, as PDAs were largely discontinued after the extensive adoption of smartphones in the early 2010s, resulting in patient monitoring and other devices becoming less popular and attractive in health care compared with smartphones [39-41]. As a result, recent publications in the mHealth domain are mostly related to smartphone-based interventions, and it remains unclear whether it is advantageous to include outdated devices in current reviews [42]. Third, most of the published studies on smartphone-based interventions include exclusively adults, despite the importance of PA levels during adolescence. Finally, and most importantly, current reviews do not provide an exhaustive review of systematic practices and methods along the trajectory of the development and evaluation of smartphone-based mHealth interventions for PA promotion, as they mostly focus on reviewing specific aspects of interventions, such as effectiveness and validity. Overall, these reviews lack a clear representation of the mHealth PA development trajectory and the tools available for researchers along this trajectory (eg, taxonomies, theories). We argue that a clearer understanding of these would significantly improve the quality of development, end product, and reporting of mHealth interventions and would contribute to the development of *theory-based* rather than *theory-inspired* interventions.

### Goal of This Review

This scoping review addresses these issues. It includes studies describing or evaluating smartphone apps alone or in combination with wearables as a primary intervention component to enhance PA levels, focusing on studies with healthy individuals without chronic or acute conditions (excluding cardiovascular diseases and obesity), and targeting studies with PA as a primary outcome. Although we included all age groups to provide a comprehensive review, we focused on one part of the analysis on studies involving adolescents, as the biggest impact on future generations’ health is to be expected from changing their behavior. The primary objective of this scoping review is to *analyze the development and evaluation trajectory of mHealth PA interventions* and to *review* *systematic theory- and evidence-based practices and methods* that are implemented along this trajectory. The secondary objective of this review is to *identify the range of evidence (both quantitative and qualitative) available on smartphone-based mHealth PA interventions* to provide a comprehensive tabular and narrative *review of the available literature* in terms of its nature, features, and volume.

This review is guided by the following research questions: (1) What kind of literature is available in the field, and how can the existing literature be categorized? (2) Which theories and techniques are implemented in smartphone-based PA interventions to support behavior changes, and how are these theories and techniques systematized? (3) Which practices and methods are used to systematically develop and evaluate smartphone-based PA interventions? and (4) Which devices and primary outcomes are used for data collection and analysis in smartphone-based PA interventions?

## Methods

### Study Design

Methodological guidelines for scoping reviews developed by Arksey and O'Malley [43], extended by Levac et al [44] and Peters et al [45], were accommodated, and the methodology adopted by McCallum et al [32] was implemented. In accordance with these guidelines, studies were not rejected based on their research design or quality.

### Identification of Relevant Articles

The literature search was conducted from September 2017 to August 2018 in three databases: MEDLINE/PubMed, ScienceDirect, and ResearchGate. The search was limited to studies published in 2008 and later, as Apple App Store and Google Play (formerly known as the Android Market) started in July and October of that year. Only publications in English were considered. Full papers, study protocols, conference proceedings, dissertations, and books were considered eligible. Reference lists of germane articles and review studies were manually searched to identify potentially relevant articles. The articles were initially screened by the first author (AD). As per best review practice, an assistant reviewer independently reviewed the eligibility of articles for inclusion in the review. Inconsistencies were resolved by discussion and consensus between the 2 reviewers.

Search strategies for MEDLINE/PubMed were developed using a combination of thesaurus and free terms based on Boolean logic (Table 1).

**Table 1:** Search builder for MEDLINE/PubMed.

Search lines	Search terms	Filtered by
Line 1	mobile phone OR cell phone OR smartphone OR smart phone OR smart-phone OR mobile device OR iphone OR mobile technology OR mhealth OR android	Title or abstract
2. AND	app OR apps OR application OR intervention OR trial OR behavior OR behaviour	Title or abstract
3. AND	physical activity OR exercise OR fitness	Title or abstract
4. NOT	heart attack OR heart failure OR cancer OR diabetes OR diabetic OR injury OR injuries OR alcohol OR sexual OR e-learning OR home OR HIV OR pain OR sleep OR smoke OR smoking OR epileptic OR rehabilitation OR asthma	Title

The use of this search builder was not possible for ResearchGate and ScienceDirect. Consequently, various combinations of the following search terms were used: *mobile phone*, *cell phone*, *smartphone*, *smart phone*, *smart-phone*, *mobile device*, *iphone*, *mobile technology*, *mhealth*, *android*, *app*, *apps*, *application*, *intervention*, *trial*, *behavior*, *behaviour*, *physical activity*, *exercise*, and *fitness*.

To select articles that were related to mHealth interventions with the primary outcome in PA, the following terms were used to manually filter out articles from the initial search results: *weight*, *eat*, *nutrition*, *diet*, and *game*.

### Study Selection

Although all age groups were included, an additional subanalysis for adolescents’ target groups was conducted (specifically accounting for BCTs effective for this target population). This was also done to contrast the differences in BCTs used in adolescents and other target populations. Studies were included if (1) the primary component of the intervention involved a mobile app targeting PA and SB and (2) the study used smartphones with available embedded sensors alone (stand-alone intervention) or in conjunction with other external components, for example, accelerometers, pedometers, and websites accessed through desktop computers (multicomponent interventions). Studies were excluded from the review if (1) the intervention was limited to using text messages only, (2) the app was used for data collection only (eg, phone-based questionnaires), (3) the intervention included any mobile device other than smartphone or PA tracker, for example, PDAs, (4) the intervention targeted other preventive health issues, such as alcohol abuse, smoking, and sport injuries, and (5) they focused on patients with chronic conditions other than cardiovascular diseases and obesity, for example, diabetes mellitus. This review includes experimental and descriptive studies, as well as reviews addressing smartphone-based mHealth interventions aimed at promoting PA. Two additional groups of studies were included: reviews or content analyses of PA trackers and meta-analyses exploring BCTs and their efficacy. This approach was used to obtain additional evidence from the domains, which are closely related to smartphone-based mHealth PA promotions, to provide theoretical evidence related to the field and to present the latest developments in the domain. Instead of considering studies using combined interventions designed to reduce body weight (ie, PA promotion and dietary interventions), we aimed to include studies promoting PA and reducing sedentary time, as it is difficult to disentangle the effects of specific intervention BCTs on particular behaviors in studies targeting several health behaviors. For example, a BCT such as *adding objects to the environment* as a part of one intervention may be successful in terms of changing eating behaviors, while having a neutral or even negative effect on PA outcomes. Therefore, we tried to avoid drawing conclusions on the effectiveness of BCTs across interventions targeting PA behavior only and interventions targeting PA, eating, and other behaviors.

### Data Extraction, Collation, Summary, and Reporting of Results

A data extraction form was developed specifically for this review and served as a basis for Tables S1-S10 presented in Multimedia Appendix 1 [2,22,24-30,35-37,46-181]. A mixed methods descriptive approach was adopted to analyze the extracted data [32]. The identified articles were categorized into 10 groups: commercial smartphone app content analyses, smartphone-based intervention review studies, activity tracker content analyses, activity tracker review studies, meta-analyses of PA intervention studies, smartphone-based intervention studies, qualitative formative studies, app development descriptive studies, qualitative follow-up studies, and other related articles.

For all groups of publications, data were extracted for author, year, target group, and targeted behavior. Depending on the group, data were further extracted for several additional categories, as follows:

- For commercial smartphone app content analyses, data were extracted for evaluation framework or taxonomy used for coding, number of apps included, app market name and category, and findings related to the theoretical background.
- For smartphone-based intervention review studies, data were extracted for taxonomy used for coding, information on BCTs, identified psychological theories, number of studies included, objective, industry-recognized reporting guidelines.
- For activity tracker content analyses, data were extracted for evaluation criteria or taxonomy used for coding, number of trackers included, number of BCTs included (mean value), BCTs present in all included devices, and BCTs present in none of the included devices.
- For activity tracker reviews, data were extracted for evaluation criteria or taxonomy used for coding, number of studies included, industry-recognized reporting guidelines.
- For meta-analyses, data were extracted for taxonomy used for coding, BCTs associated with more effective interventions, BCTs associated with less effective interventions, and industry-recognized reporting guidelines.
- For smartphone-based intervention studies, data were extracted for pilot, protocol, sample size, theoretical background, study design, study duration, stand-alone or multicomponent intervention, principal outcome measures, industry-recognized reporting guidelines, and PA recommendations.
- For qualitative formative studies and qualitative follow-up studies, data were extracted for sample size, theoretical background, and method of data collection.
- For app development descriptive studies, data were extracted for sample size, theoretical background, commonly reported systematic theory or evidence-based practices, and methods for development, evaluation, and reporting.
- For all other related articles, data were extracted for keyword, title, type of study or methodology, and objective and narratively described further.

## Results

### Summary of Search Results

**Figure 1:**
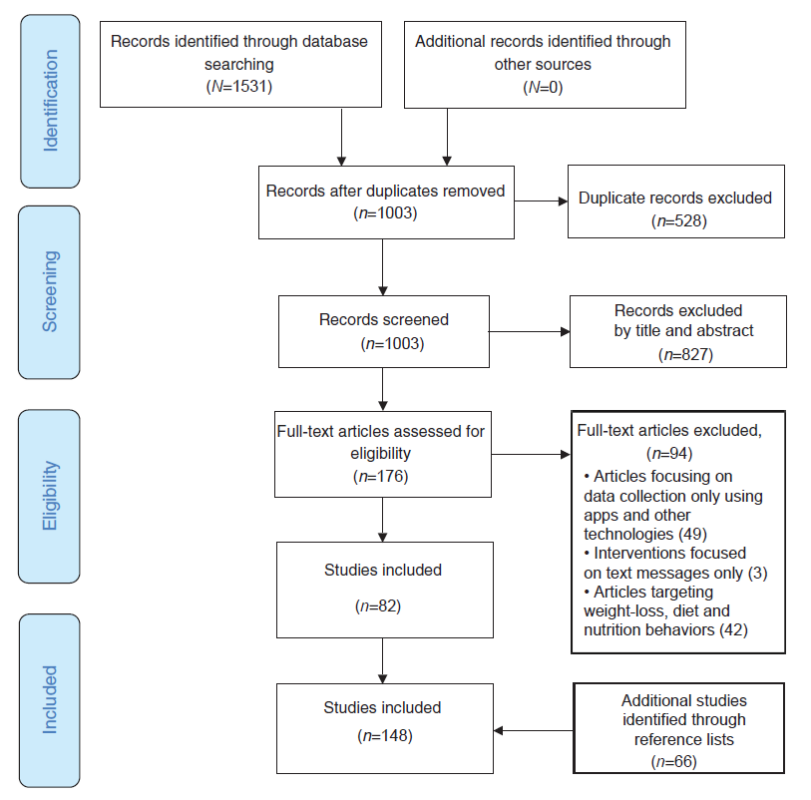
PRISMA (Preferred Reporting Items for Systematic Reviews and Meta-Analyses) flow diagram.

A total of 1531 articles were identified during the initial database search. The searches of the MEDLINE and PubMed and ScienceDirect databases yielded 785 and 546 results, respectively. ResearchGate database search results were restricted to 200 because the database search engine generated an unlimited number of search results. After the removal of duplicates, 1003 articles were screened for their titles and abstracts, resulting in 176 full-text articles. Of these, 94 full-text articles were excluded for the following reasons. The resulting 82 articles were hand-searched for references to relevant articles, leading to the identification of an additional 66 articles. As a result, 148 articles were included in the review (Figure 1).

### Categorization of the Literature Available in the Field

**Figure 2:**
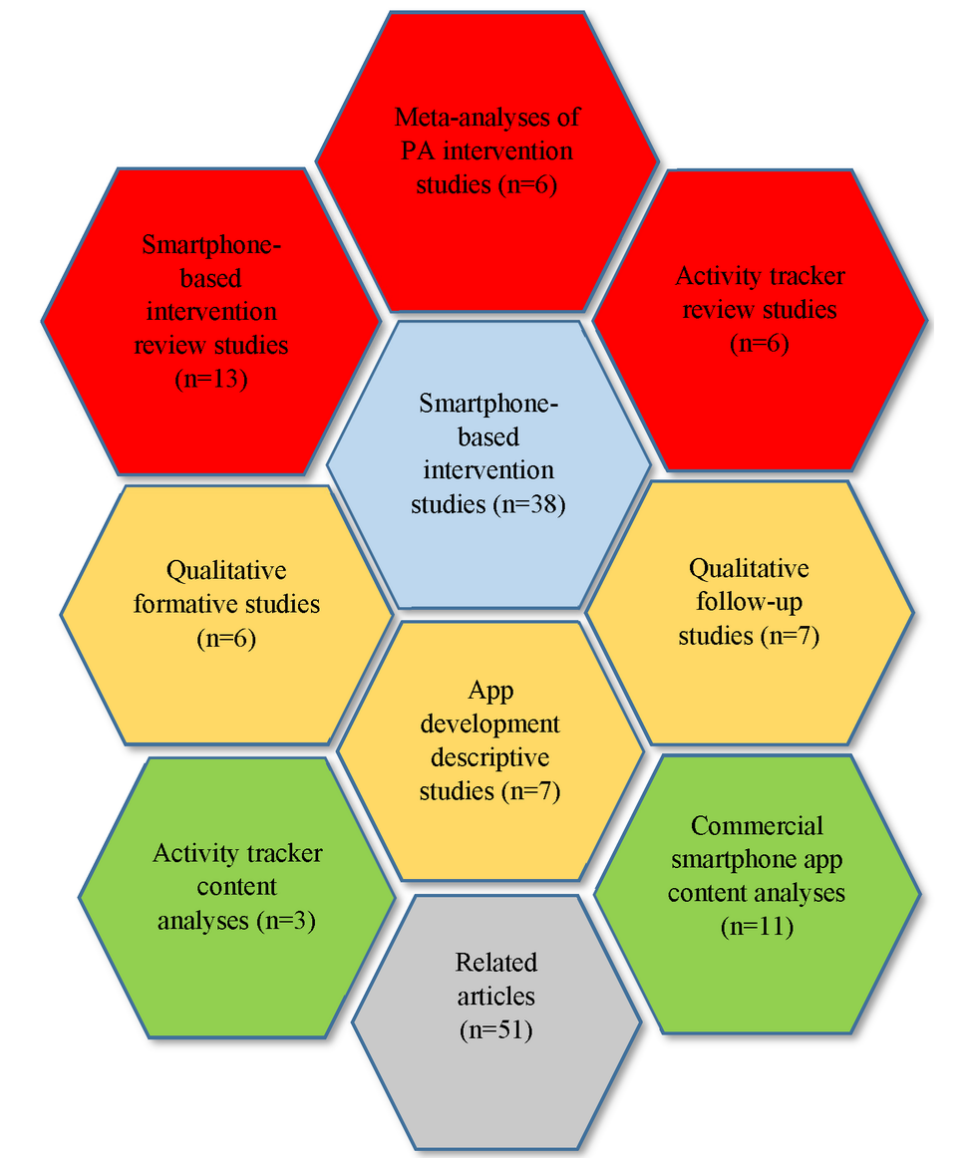
Map of search results by number of studies. PA: physical activity.

To categorize the included studies, we used the stepwise approach developed by Whittaker et al [182]. Whittaker et al [182] organized the study methods according to the research and evaluation steps in the development of an mHealth intervention. We mapped the identified studies in the same fashion, aligning the development and evaluation trajectory of mHealth PA interventions with the study types used along this trajectory. Although the approach developed by Whittaker et al [182] was the most fitting, it did not accommodate all the identified study types; therefore, a more fine-graded stepwise trajectory was developed. After the literature search was completed, 148 included studies were divided into 10 groups according to the study type (Figure 2), which was in line with the adopted development and evaluation trajectory. If a study could not be allocated to one specific category, it was included in the group *Related Articles* section. After conducting the analyses of the included studies, commonly reported systematic theory or evidence-based practices and methods for development, evaluation, and reporting of mHealth PA interventions were identified (Multimedia Appendix 1). This was the categorization principle used in this review.

To improve further categorization attempts, we refined the outcome of our analysis, which resulted in the table presented below (Table 2). This categorization system may be advantageous for future studies.

**Table 2:** Possible categorization approach for smartphone-based interventions for physical activity promotion.

Steps in the development and evaluation process [182] and development and evaluation trajectory of mHealth^a^ PA^b^ interventions, study type					Common reported systematic theory or evidence-based practices and methods for development, evaluation, and reporting		Purpose
**Formative research**							
	**Summarizing findings**						
		Commercial smartphone app content analyses		BCT^c^ taxonomies Scales to assess app quality PA recommendations		To critically evaluate the material that has already been published To provide an overview of the current state of knowledge	
		Smartphone-based intervention review studies		Industry-recognized reporting guidelines Behavior change theories or models BCT taxonomies			
		Activity tracker review studies		Industry-recognized reporting guidelines			
		Activity tracker content analyses		BCT taxonomies			
	**Synthesizing findings**						
		Meta-analyses of PA intervention studies		BCT taxonomies Industry-recognized reporting guidelines		To assess the strength of evidence present through establishing statistical significance	
	**Qualitative formative research**						
		Qualitative formative studies (assessing general topic perception by target users)		Mixed methods data collection (surveys, questionnaires, interviews, and focus groups discussions)		To inform the development of the intervention	
**Pretesting**							
	**Describing an intervention**						
		App development descriptive studies		BCT taxonomies Behavior change theories or models PA recommendations Scales to assess app quality		To describe the intervention development process and intervention features To control acceptability, engagement, and experiences of proposed intervention to target audience To improve and refine intervention on the basis of qualitative feedback	
**Pilot study**							
	**Pilot testing**						
		Pilot trials		Behavior change theories or models PA recommendations Industry-recognized reporting guidelines Trial designs (RCTs^d^, experimental trials, and rapid design trials)		To examine content of intervention To examine feasibility of a trial approach, trial processes (eg, recruitment, registration, data collection), methods	
	**Trial protocol**						
		Study protocols		Behavior change theories or models PA recommendations Industry-recognized reporting guidelines Trial designs (RCTs, experimental trials, and rapid design trials)		To describe processes of trials (eg, recruitment, registration, data collection)	
**RCTs**							
	**Testing**						
		Clinical trials		Behavior change theories or models PA recommendations Industry-recognized reporting guidelines Trial designs (RCTs, experimental trials, and rapid design trials)		To examine the effect of the intervention as a whole package or the effect of one of its components	
**Qualitative follow-up**							
	**Qualitative follow-up evaluation**						
		Qualitative follow-up studies (assessing the developed intervention by target users)		Mixed methods data collection (surveys, questionnaires, interviews, and focus groups discussions) Behavior change theories or models		To control acceptability, engagement, and experiences of proposed intervention to target audience To control implementation issues To control the effect of the intervention after dissemination	

^a^mHealth: mobile health.

^b^PA: physical activity.

^c^BCT: behavior change technique.

^d^RCT: randomized controlled trial.

### Study Characteristics

#### Overview

The characteristics of the included studies are presented in Tables S1-S10 of Multimedia Appendix 1. All included articles (n=148) were separated according to the subject of the article in the following groups: commercial smartphone app content analyses (n=11), smartphone-based intervention review studies (n=13), activity tracker content analyses (n=3), activity tracker review studies (n=6), meta-analyses of PA intervention studies (n=6), smartphone-based intervention studies (n=38), qualitative formative studies (n=6), app development descriptive studies (n=7), qualitative follow-up studies (n=7), and related articles (n=51). All articles were published between 2008 and 2018. The most common targeted behaviors were PA, SB, and dietary behavior, although the majority of the included articles targeted a single health behavior, namely PA. Although the majority of studies included adult populations (125), 24 articles targeted children and adolescents (age range: 5-19 years).

#### Commercial Smartphone App Content Analyses

Articles were allocated to this group if the objective of the study was to analyze the content of commercial apps presented on digital distribution platforms (ie, App Store, Google Play, and Microsoft Store). The included studies (n=11) were published from 2012 to 2018, and most of them targeted the general population (n=7) and adults (n=2), whereas only 2 targeted children and adolescents. More than half of the content analyses targeted PA behavior (n=7); the other reported lifestyle-related health behaviors, outcomes and aims were SB, diet, health and fitness, and obesity prevention. Sample sizes ranged between 25 and 3336 (mobile) apps, and the most common digital distribution platform was App Store (n=11). A total of 6 studies used different variations of the BCT taxonomy (26, 40, and 93 BCTs) as an evaluation or coding framework. The average number of the BCTs in those studies ranged from fewer than 4 to 8.1, and the most common BCTs for adults included *provide instruction*, *provide feedback on performance*, *prompt specific goal setting*, *prompt self-monitoring of behavior* (26 BCTs taxonomy [35]), *provide instruction on how to perform the behavior*, *provide feedback on performance*, *goal setting (behavior)*, *prompt self-monitoring of behavior* (40 BCTs taxonomy [36]), *instruction on how to perform the behavior,* *feedback on behavior*, *goal setting (behavior*), and *self-monitoring of behavior* (93 BCTs taxonomy [37]). Interestingly, this supports the study reporting that the average number of BCTs used in gamified apps aimed at health promotion was higher (14 BCTs) [46] than in nongamified health promotion apps. For children and adolescents, only one study reported the most frequently used BCTs [47]. They were *providing instructions*, *general encouragement*, *contingent rewards*, and *feedback on performance* (26 BCTs taxonomy). The two most recent studies [47,48] used the Mobile App Rating Scale (MARS) to assess the quality of apps. On a 5-point scale, the overall app quality was moderate: the total MARS score ranged from 3.6 to 3.88 points.

#### Smartphone-Based Intervention Review Studies

This group included intervention studies aimed at reviewing smartphone-based intervention publications. The included reports (n=13) were published between 2013 and 2017 and targeted the general population (n=6), adults (n=4), and children and adolescents (n=3). More than half of the reviews targeted PA behavior exclusively (n=7), whereas the other reported lifestyle-related health behaviors and outcomes and aims were SB, diet, weight reduction, obesity combatting, healthy nutrition, and overweight prevention. The number of articles included in these reviews ranged from 7 to 52. Only two studies used the taxonomy of BCTs (26 and 93 BCTs) to code the included interventions [49,50]. These studies reported that for adults the following BCTs were most frequently employed: *goal setting (behavior)*, *self-monitoring of behavior*, *social support (unspecified)*, *feedback on behavior*, *instruction on how to perform the behavior*, *adding objects to the environment*, *information about health consequences*, and *prompts or cues* (93 BCTs taxonomy). For adolescents, *prompt self-monitoring of behavior* and *provision of feedback on performance* techniques were most often applied (26 BCTs taxonomy). The other 4 studies provided information about behavioral components without mentioning any taxonomy used for coding [24,29,51,52]. Self-monitoring, cues to action, feedback, and social support were identified as the most commonly used BCTs [29]. The most efficacious and helpful BCTs were reported to be goal setting, self-monitoring, performance feedback, motivational cuing, rewards, social support, and coaching [24,51,52]. The majority of the identified reviews (n=9) reported the theoretical background of smartphone-based interventions. The most frequently used theoretical framework was the Social Cognitive Theory (n=7), followed by the transtheoretical model (n=4), Self-Determination Theory (n=4), and the Theory of Planned Behavior (n=2). The other reported models and theoretical approaches included the Persuasive Systems Design Model, the Control Systems Theory of Self-regulation, the Behavior Change Wheel, the Five A’s Model, the Fogg Behavior Model, Learning Theory or operant conditioning, Social Influence Theory, the Theory of Reasoned Action, and Cognitive Behavior Therapy. Of 13 reviews, 3 used the PRISMA (Preferred Reporting Items for Systematic Reviews and Meta-Analyses) reporting guidelines [24,25,50].

#### Activity Tracker Content Analyses

Articles were included in this group if the objective of the content analysis was to analyze the theoretical components included in the activity trackers. The included studies (n=3) were published between 2014 and 2017 and targeted the general population [53-55]. The majority of the content analyses targeted PA behavior exclusively (n=2), whereas the other reported lifestyle-related health behaviors were SB and sleep. The number of included activity trackers per article ranged from 3 to 13. All 3 studies used the taxonomy of BCTs (40 and 93 BCTs) to code the included interventions [53-55]. According to these content analyses, the average number of BCTs included in the activity monitors ranged between 9 and 25 BCTs (40 BCTs taxonomy). There was an agreement between 2 studies about BCTs present in all included devices, which were *provide information about others’ approval*, *provide normative information about others’ behavior*, *prompt review of behavioral goal*, *provide rewards contingent on successful behavior*, *prompt self-monitoring of behavior*, *prompting focus on past success*, *provide feedback on performance*, *facilitate social comparison*, and *plan social support or social change* (40 BCTs taxonomy) [53,54]. According to the same studies, *prompt anticipated regret*, *fear arousal*, *prompt self-talk*, *prompt use of imagery*, and *general communication skills training* BCTs were not present in any of the included devices (40 BCTs taxonomy).

#### Activity Tracker Review Studies

Review studies in this group aimed to provide evidence on the effectiveness, efficacy, feasibility, validity, or reliability of activity trackers. The included studies (n=6) were published between 2012 and 2018 and targeted adults (n=5) and children and adolescents (n=1) [56-61]. The majority of the reviews targeted PA behavior exclusively (n=5), whereas the other reported lifestyle-related health behavior was sleep. The number of articles included per review ranged between 5 and 134 publications. Five studies used PRISMA or PRISMA-P (Preferred Reporting Items for Systematic Reviews and Meta-Analyses Protocols) reporting guidelines [56-60].

#### Meta-analyses of PA Intervention Studies

Articles were included in this group if the objective of the meta-analysis was to analyze PA intervention studies and to define the BCTs that were associated with more or less effective interventions. It is important to note that all identified meta-analyses (n=6) reviewed only *classic* interventions and did not include smartphone-based interventions [62-67]. Only one meta-analysis reviewed PA smartphone-based interventions [50]. However, this meta-analysis was excluded because it did not provide information on the effectiveness of BCTs because of the small number of included studies.

Articles included in this group were published between 2009 and 2017 and mainly targeted adults with one exception, where the targeted group included children and adolescents [65]. All the included meta-analyses targeted PA, with 3 studies additionally targeting healthy eating (HE) and diet [62,65,67]. Two studies used 26 BCTs taxonomy for coding, 3 studies used 40 BCTs taxonomy for coding, and 1 study used the latest 93 BCTs taxonomy for coding. Reported results for BCTs associated with interventions that are more effective were divergent, with *self-monitoring* and *feedback* reported to be effective according to 4 and 3 meta-analyses, respectively. Every meta-analysis reported different results for BCTs associated with less effective interventions. For the adolescent target group, BCTs (26 BCTs taxonomy) associated with more effective interventions include *provide information on consequences*, *provide information about others’ approval*, *prompt intention formation*, *prompt self-monitoring of behavior*, and *agree on behavioral contract* [65]. The *provide instruction* BCT was associated with less effective interventions in adolescents [65]. Only the latest meta-analysis used PRISMA reporting guidelines [67].

#### Smartphone-Based Intervention Studies (Study Protocols, Pilot Trials, and Clinical Trials)

The smartphone-based intervention study group included 38 articles representing 32 research studies published between 2008 and 2018. The majority of these studies targeted adults (n=20), whereas 12 targeted adolescents, and the sample size ranged from 8 to 700 participants, and the duration of interventions ranged from 2-32 weeks (most common duration: 8 weeks). The participants’ ages ranged from 8-81 years. A total of 14 studies exclusively targeted PA behavior; the other reported lifestyle-related health behaviors, aims, concepts, outcomes, and conditions included weight loss, SB, cardiorespiratory fitness, diet, sleep, fitness, and obesity. The most common study design was a two-arm randomized controlled trial (RCT; n=10); for other study designs, the number of intervention groups ranged between 1 and 4. There was a preponderance in the number of multicomponent interventions (n=19) over stand-alone interventions (n=13). The interventions mainly used newly designed smartphone apps (n=29) rather than commercially available apps (n=3), the theoretical background of which was unknown. The most common outcome measures were minutes spent with moderate-to-vigorous PA (MVPA) and a daily step count. In total, 14 studies did not report a theoretical background. For adults, the most frequently used theoretical framework was Social Cognitive Theory (n=11), followed by Self-Regulatory Theory (n=3) and the Fogg Behavior Model (n=2). Of the 12 studies including adolescents, several (n=4) did not report any theoretical background, and among those who did, Self-Determination Theory (n=6) was the most frequently used. The other reported theoretical frameworks and models include the Theory of Meaning Behavior, the Five Factor Model of Personality, the Health Belief Model, the Technology Acceptance Model, the Theory of Motivation in Videogames, the Transtheoretical Model of Health Behavior Change, the Functional Triad, the Transcontextual Model of Motivation, the Synergy Hypothesis, Learning Theory, Basic Psychological Needs Theory, the COM-B (Capability, Opportunity, Motivation, Behaviour) model, and the Behavior Change Wheel. A total of 10 studies used CONSORT (Consolidated Standards of Reporting Trials) reporting guidelines, 1 study used SPIRIT (Standard Protocol Items: Recommendations for Interventional Trials) reporting guidelines, and 2 studies used both CONSORT and SPIRIT reporting guidelines.

#### Qualitative Formative Studies

Articles in this group used a qualitative approach to examine users’ views of and preferences for app features in terms of usability and attractiveness, among others, that can inform the development of future mHealth PA interventions. The identified studies (n=6) were published between 2011 and 2016 and included adults (n=5) and adolescents (n=1) target populations [68-73]. Sample sizes ranged between 14 and 120 participants, and research designs included focus groups (n=3), web-based surveys (n=3), and individual interviews (n=3). The studies focused on the perception of apps targeting PA (n=3), health behavior change in general (n=2), and health and fitness (n=1). The app features that were evaluated by participants in the majority of these studies (n=4) were social networking, context sensing or personalization, design, self-monitoring, and goal setting.

Social networking, that is, exposing one’s health behavior through integration of the PA app in social networks (eg, Facebook), was generally perceived negatively. Context sensing or personalization, self-monitoring, and goal setting were perceived as valued features in smartphone apps. The design of the app appeared to be a crucial feature, in that users preferred a simple and structured layout, which was easy to use, playful, and fun. Apps were not used or uninstalled if they contained unnecessary features, required excessive data entry for sign up, had complicated operating procedures, and required instructions that were time-consuming or burdensome.

#### App Development Descriptive Studies

Articles were included in this group if the objective of the study was to describe the intervention development process and intervention features. The identified studies (n=7) were published between 2012 and 2018 and included adults (n=5), adolescents (n=1), and general populations (n=1) [74-80]. The intervention groups differed in terms of sample size, ranging from 10 to 68 participants. The developed interventions mostly targeted PA (n=6), and other related behaviors, such as SB (n=2) and weight loss (n=1). Common reported systematic theory or evidence-based practices and methods for development, evaluation, and reporting included PA recommendations (n=1) [76], BCT taxonomies (n=2) [78,80], and MARS (n=1) [79]. The most frequently used theoretical framework was Social Cognitive Theory (n=5); the other reported theoretical frameworks and models included Health Belief Model, Theory of Planned Behavior, Technology Acceptance Model, Fogg Behavior Model and Self-Determination Theory.

#### Qualitative Follow-up Studies

This group of studies aimed at assessing the acceptability, engagement, and experiences of the target audience with the intervention and the effect of the intervention after dissemination. The identified studies (n=7) were published between 2012 and 2017 and included adults (n=4) and adolescents (n=3) as target populations [81-87]. Sample sizes ranged between 5 and 68 participants and research designs included surveys (n=3), interviews (n=2), focus groups (n=1), and questionnaires (n=1). The apps included in the studies targeted PA (n=5), fitness (n=1), and well-being (n=1). The theoretical frameworks and models were reported only in 2 studies, which included the Theory of Planned Behavior in both studies (n=2) [84,87]. The other reported theoretical frameworks and models included the Theory of Meaning Behavior, the 5 Factor Model of Personality, and the Functional Triad [84,87].

#### Related Articles

The related articles group included 51 articles published between 2008 and 2018. These articles were mainly identified through manual reference searches, and although they were relevant to the topic of this review, they did not fit into the other groups presented above. The study types included methodological, theoretical, conceptual studies; reports; recommendations from workshops; other literature reviews (reviews of methodological, theoretical, and conceptual studies); and reviews and trials on related topics (eg, gamification) that represented theoretical and methodological findings and recommendations that were grouped into several topical subgroups: activity tracking, automation, BCT, behavior change theory, GPS, just-in-time adaptive interventions, mHealth apps, PA, profiling, and RCT alternatives for mHealth. Relevant information from these articles was analyzed and presented narratively in the *Discussion* section.

## Discussion

### Theories and Techniques Implemented in Smartphone-Based PA Interventions to Support Behavior Changes: Current Situation and Recommendations

The science of behavior change has advanced significantly in recent years. Nevertheless, many challenges remain concerning the standardization of the development and reporting of methods of behavior change interventions. As presented in the tables of Multimedia Appendix 1, there is a plethora of approaches in developing smartphone-based PA interventions; however, most of them have been developed and reported without an explicit theoretical foundation. This has been described as the development of *theory-inspired* interventions (in which the theoretical background is often chosen depending on the experiences and preferences of researchers and developers), rather than *theory-based* interventions (in which the chosen theoretical background was measured and tested in the intervention or conditions) [88].

To accomplish a more standardized methodological approach, several frameworks have been developed by Michie et al [88] in the domains of smoking, PA and HE, alcohol consumption, and safer sex. For the PA and HE domains, these authors developed the *Behavior Change Technique Taxonomy*, in which a BCT is defined as “an observable and replicable component designed to change behavior” [37]. It is the smallest component compatible with retaining the postulated active ingredients and can be used alone or in combination with other BCTs [89]. The taxonomy itself has been described as “an extensive, integrated, hierarchical classification system for reliably specifying intervention components (BCTs)” [88]. There are three versions of the BCT taxonomy: *A Taxonomy of Behavior Change Techniques* (26 BCTs), developed for coding PA and HE interventions for adults in 2008; *The CALO-RE (Coventry, Aberdeen & London–Refined) taxonomy* (40 BCTs), developed in 2011, which is the extended version of the previous taxonomy; and finally *The Behavior Change Technique Taxonomy (v1)*, which is the latest (developed in 2013) 93 BCTs cross-domain taxonomy, and which is recommended to be used instead of the previous versions, which are considered as “domain specific proto-versions” [35-37,89].

In addition, Michie et al [88,183] created a compendium of 83 theories of behavior and behavior change, containing more than 1700 theoretical constructs, some of which can be potentially considered as so called “theoretical mechanisms of action.” In this context, mechanisms of action are conceptualized as “a range of theoretical constructs, defined broadly as the processes through which a behavior change technique affects behavior” [88].

To overcome the unsystematic intervention development and reporting, it is also important to understand how BCTs can be linked to theoretical mechanisms of action, which is currently being investigated [88,90]. Such a link will provide a basis for a systematic and transparent method for developing behavior change interventions. Until then, the *Behavior Change Wheel* was considered the most appropriate development framework for selecting appropriate BCTs for specific behavior change interventions. This framework was also developed by Michie et al [91,184], introducing a synthesis of 19 behavior change frameworks, providing a systematic guide for designing and evaluating behavior change interventions and policies.

Several important tendencies were identified in all the included groups of studies. First, studies aiming to promote PA via smartphone-based interventions in adolescents are underrepresented in comparison with those targeting adults. While analyzing the studies including adolescents, Schoeppe et al [24] confirmed that there was no difference in the BCTs incorporated in apps for adolescents compared with those used in apps for adults. This is surprising, as adolescents’ motivations, social environment, and financial opportunities, among others, are much different from those of adults [185].

Second, the tables in Multimedia Appendix 1 demonstrate that there is no agreed evaluation framework or taxonomy to code or report smartphone-based PA interventions. Researchers did not state the coding methods [24,29,51,52], used various evaluation frameworks [92-94], or used different versions of the BCT taxonomy by Michie et al [66,89], who developed all versions of the BCT taxonomy, recommend using the latest version, which consists of 93 BCTs, and although several authors justified their preference for the specific version of the taxonomy (eg, O’Brien et al [66] stated that the 40 BCTs CALO-RE taxonomy was used “as it was specifically developed for use with PA and dietary interventions”), it is evident that such an approach is disadvantageous because it hinders the systematic accumulation of evidence. However, this is not surprising, as the field of mHealth is still a fairly young field of research, where new, dynamic theories and models of behavior that better fit the capabilities of mobile systems have yet to be developed or are currently under development [186]. These new developments in behavioral models and mechanisms of action must be taken into account for the field to progress. Thus, while striving for uniformity in reporting, researchers should periodically *upgrade* their reporting methods while maintaining a balance between systematic and innovative approaches. It is therefore important to realize that as the field grows, the taxonomy will be extended and modified, and it will be subject to further refinement and development, as stated by Michie et al [37].

Third, the tables in Multimedia Appendix 1 demonstrate that there seems to be no consensus about the optimal behavior change theory or model that should be used in smartphone-based interventions for PA promotion. Until now, there has been no clear evidence for the best behavior change model; however, the results show that Social Cognitive Theory seems to be the most favored among researchers. Progress in this field of research will be hampered if theoretical models on which interventions are based are not selected according to explicit criteria but on personal preferences. Although some researchers continue in their work to eventually provide systematic solutions, the most coherent approach at this time seems to consist of selecting BCTs based on the features and goals of the designed intervention using the Behavior Change Wheel framework [88,184].

Reviews of the commercial app market (Table S1 in Multimedia Appendix 1) also suggest that there is a lack of theory-based and evidence-based apps [92,95-98]. One should differentiate those terms; although the first usually refers to the systematic selection of BCTs and theories or models, the second refers to the compliance of apps with various national and international PA norms.

As previously outlined, the included meta-analyses did not analyze smartphone-based interventions. We decided to include them here, based on the rationale of Brannon and Cushing [65], who state that *classic* and smartphone-based interventions do not represent disparate bodies of evidence. After comparing BCTs used in commercial, research-driven apps, activity trackers, and meta-analyses (corresponding tables in the Multimedia Appendix 1), it becomes clear that there is no agreed theoretical or evidence base for the choice of BCTs in smartphone-based interventions. However, it is still important to report on and verify accumulated evidence for the field to progress, while considering its inconclusive nature. For adolescents, a comparison of the review by Schoeppe [24] and Brannon and Cushing meta-analysis [65] shows that BCTs associated with effective interventions *provide information about others’ approval* and *prompt self-monitoring of behavior*.

There is a clear need to conduct meta-analyses on mHealth studies. Until now, such a meta-analysis has been conducted once by Direito et al [50]; however, the author concentrated more on RCTs of mHealth technologies, rather than smartphone-based mHealth interventions tested with more suitable trial designs. As a result, only RCTs were selected for this meta-analysis, despite the latest considerations that RCTs may not provide the most advantageous design for the evaluation of mHealth interventions [99]. Second, out of the 21 studies included, only 5 described smartphone-based interventions, whereas the rest included interventions delivered through a website, SMS text messages, and PDA devices, that is, modes of delivery that are often considered outdated in the mHealth domain.

In general, it is also important to consider the mechanisms of action and the parameters of effectiveness of coded BCTs. Although the current approach applied for coding, using the taxonomy of Michie et al [37], does not consider the context of BCTs, Kok et al [100] argued that this is crucial. They state that the taxonomy developed by Michie et al [37] is useful for coding, but is not a good basis for intervention development, as it may contain ineffective and even countereffective methods (BCTs) [100]. Kok et al [100] define the *parameters of effectiveness* as “the conditions that must be satisfied in practical applications for the method (BCT) to be effective” and add that if parameters of effectiveness for the particular method (BCT) are violated, it may become less effective or even countereffective. Consequently, an alternative, that is, *A Taxonomy of Behavior Change Methods*, has been designed to take parameters of effectiveness into consideration, while developing an intervention [100]. Various researchers, including Michie et al [88], support the idea that BCTs should not be treated in a vacuum, considering their context and possible combinations [101-103].

When selecting a theoretical model, many researchers seem to assume that the basic motivation of the user is to become more physically active, which is not always the case [104]. Therefore, which models consider the level of motivation before favoring a specific framework is worth assessing [104]. Interestingly, the present analysis of intervention studies shows that only interventions targeting adolescents used Self-Determination Theory, whereas for adults, Social Cognitive Theory was by far the most frequently used model. Finally, researchers have recently started to question whether the theoretical models developed before the invention of smartphones, and digitalization in general, are still applicable [51,105,106,186]. Such critiques are justifiable, as digital devices such as smartphones provide unprecedented opportunities for observation, data collection, and just-in-time interventions and, therefore, the interaction between the user and the device delivering the intervention [26].

### Other Commonly Reported Systematic Theory or Evidence-Based Practices and Methods for the Development, Evaluation, and Reporting of Smartphone-Based PA Interventions: Current Situation and Recommendations

#### Overview

As is evident from the results in this review and the tables presented in Multimedia Appendix 1, commonly reported systematic practices and methods could be successfully identified. They include PA recommendations, trial designs (RCT trials, experimental trials, and rapid design trials), mixed methods data collection (surveys, questionnaires, interviews, and focus group discussions), scales to assess app quality, and industry-recognized reporting guidelines. Nevertheless, there seems to be no consensus on which practices and methods are preferable to use, which reflects the same tendency as outlined for theories and techniques. To advance this field of research, researchers and developers should consider using existing practices and methods depending on the aims and features of the developed intervention. The more systematic the development process, the higher the replicability of the results. As a result of the current review, we provide a list of best practices and methods that can be used during the development evaluation and reporting of PA mHealth interventions.

#### WHO Global Recommendations on PA for Health

These are evidence-based recommendations of the WHO that “address the links between the frequency, duration, intensity, type and total amount of PA needed for the prevention of NCDs” [15]. Alternatively, researchers can use other public PA guidelines used by national agencies and health institutions, such as Canadian Physical Activity Guidelines for Adults, Physical Activity Guidelines for Americans, American College of Sports Medicine Guidelines, Center for Disease Control Guidelines, American Heart Association Guidelines, UK Department of Health Guidelines, Institute of Medicine Guidelines, and US Department of Health and Human Services Guidelines [97,98,107]. Applying one or several of these guidelines will help researchers to understand PA norms that, for instance, can be used as a PA goal for participants or inclusion and exclusion criteria.

#### MARS and User Version of the MARS

The MARS scale has been developed quite recently in many of the most recent mHealth research studies [47,79,108]. This is a “reliable, multidimensional measure for trialing, classifying, and rating the quality of mobile health apps” [109]. The MARS scale is useful if the researcher wishes to reliably rate or see the possible flaws of the developed mobile app.

#### Industry-Recognized Reporting Guidelines

The following industry-recognized reporting guidelines have been illustrated:

- PRISMA: this is an evidence-based minimum set of items for reporting in systematic reviews and meta-analyses [187].
- PRISMA-P: this is a set of items aimed at facilitating the development and reporting of systematic review protocols [188].
- CONSORT statement: this internationally acknowledged tool can be used to assess the quality of RCT studies and to design or report an RCT of the highest quality and standard [189].
- SPIRIT: a guideline for minimum content of a clinical trial protocol [190].

#### Rapid Design Trials

Although RCT study designs are widely considered a *gold standard* for intervention research in many areas, it has been suggested that they may not be the best approach for the evaluation of mHealth interventions for several reasons. First, the duration of the completion of an RCT is long (5.5 years on average from recruitment to publication of the trial results [99,110]), which in the modern ever-developing digital world may be the cause for an app becoming obsolete. Second, an RCT is a rigid design requiring interventions to remain unchanged and stable during the entire duration of the trial. This creates a problem, as software is meant to change, progress, evolve, and adapt to its user in short periods [32,51,99,108,110-114]. Therefore, mHealth interventions could make use of flexible evaluation designs and methodologies, providing timely information and being responsive and agile. Consequently, alternative designs and methodologies for the evaluation of mHealth interventions have been proposed [32,99,108,110-113]:

1. Continuous Evaluation of Evolving Behavioral Intervention Technologies
2. Sequential Multiple Assignment Randomized Trial
3. The Multiphase Optimization Strategy
4. Microrandomized trial (MRT)
5. Step-wedge design (ie, cluster randomized design)
6. n-of-1 trials
7. Practice-Based-Evidence methodology
8. Trial of Intervention Principles framework
9. Collaborative Adaptive Interactive Technology framework

However, these designs have rarely been implemented. According to the most recent review of PA apps, only 2 of 111 included studies used rapid research designs [32]. The methodology of the most recent rapid design, MRT, is currently being developed, and the first protocols and trials have been recently published [115,191].

#### Qualitative Studies

Although there is no one recommended methodology, the most commonly reported methods in identified studies include surveys, questionnaires, interviews, and focus group discussions. On the basis of this review, we cannot recommend any specific method, yet there is a clear need for more systematic reporting of results. Nevertheless, the studies summarized in Tables S7-S9 in Multimedia Appendix 1 provide some indication of the most efficacious and user-attractive features in mobile apps aimed at PA promotion.

1. Design simplicity: Ease of use and navigation through the app, absence of unnecessary features, unambiguous information, and a structured layout were all listed as features that positively affected participants’ engagement. Apps with excessive data entry for sign up, presenting features that required instructions, and complicated operating procedures were negatively perceived by users [28,29,69-71,81,82,116].
2. Personal approach for each user- tailored coaching, goals, feedback, and notifications: Users perceive a personalized approach as an important factor for motivation and engagement. Therefore, it is important to consider sociodemographic user differences [117,118]*.* Moreover, the users themselves prefer to be in control of the app’s features, having the ability to hide or add them [28,69,71,72,81,82,116].
3. Reward: A transparent reward system was positively recognized by users [28,71,82,116].
4. Self-monitoring and goal setting: These app features were the key features enjoyed or rated positively by app users [68,70,72,82].
5. Gamification: This feature can positively affect user engagement by bringing more enjoyment to exercise or activity [119,120].
6. Social networking: This feature was perceived differently in various apps:
  - *Peer-to-peer influence* was delivered through encouragement, praise, and competition with the participants’ peers. As indicated by Klasnja and Pratt [121], the results presented by different researchers are inconclusive: studies report both positive and null effects [120-125].
  - *Social support from family and friends*: As the reviews show, the effect on participants depends on the behavioral goals of friends or family members: if the goals differ, the effect of social support seems to be low [121].
  - *Social modeling* (eg, tips for health-related resources from successful peers) seems to have a positive effect on participants [121].
  - *Integration with social networks* (eg, Facebook) was perceived negatively by app users [69,71,81,82].

These findings demonstrate that a chosen method of social support can significantly affect the acceptability and usefulness of the app among users. Overall, it is important to underline the necessity of pretesting the app with a specific target audience to optimally refine the app’s features and components.

### Devices and Primary Outcomes Used for Data Collection and Analysis in Smartphone-Based PA Interventions: Current Situation and Recommendations

Smartphone-based interventions can be divided into stand-alone interventions, where only the app is used and multicomponent interventions, where the app is one of several intervention components. The choice of intervention components affects the intervention outcomes, and, if a multicomponent approach is chosen, may lead to the inclusion of various devices as additional components of the intervention.

For the majority of researchers, the selection of smartphone-based intervention components depends on several factors, such as the accuracy of data collection, device compatibility with the user, and durability. As can be seen from Table S6 in Multimedia Appendix 1, all smartphone-based intervention data collection components can be divided into three groups: smartphones, commercial activity trackers, and medical-grade activity trackers. The selection of a data collection device is usually well aligned with the chosen outcome measures.

As presented in Table S6 of Multimedia Appendix 1, a stand-alone intervention that includes only a smartphone with the installed app and inbuilt accelerometer can track the most common PA outcome measures, that is, minutes spent in MVPA and SB, and daily step count [126]. These data can also be collected with a range of precision levels (depending, for instance, on the use of built-in GPS sensors, which can provide data that are more accurate) [127-129]. The drawbacks of solely using smartphones include the short battery life of the device, only moderate accuracy levels, moderate durability, and limited exposure time (the user will not usually carry the phone during certain periods of the day) [26,121].

The validation reviews presented in Table S4 of Multimedia Appendix 1 demonstrate that commercially available, usually wrist-worn activity trackers can help collect similar data with higher accuracy levels, although still in a moderate range [56,60]. In addition, some built-in sensors (eg, heart rate [HR]) can provide supplementary data and improve the accuracy of MVPA measures. They avoid most of the drawbacks of smartphone devices, as they provide a long battery life for the device, high device durability, and extended exposure time. Commercial activity trackers show good potential in the implementation of theory-based practices and improve the data collection procedure in human physiology research for both adults and adolescents [58,120,130-132,192].

Medical-grade activity trackers (hip, waist, or wrist worn), for example, ActiGraph devices, provide the highest measurement accuracy levels; however, they also have certain drawbacks. The hip and waist location can lead to low user compatibility levels and reduced exposure, whereas HR can only be measured with a wireless HR monitor [193].

Consequently, while developing PA interventions, researchers should consider these factors and choose the device according to the characteristics most suitable for their projects. It is important to note the findings of a recent review, which confirms that multicomponent interventions tend to be associated with higher intervention efficacy [24]. Although some researchers chose to use simplistic outcome measures such as a daily step count, studies show that a multidimensional approach with several outcome measures is more comprehensive [107].

### Advancing mHealth Further: Technological Advances Applied in Smartphone-Based mHealth Interventions

Researchers working in the smartphone-based mHealth field often face problems with participants’ engagement: the long-term retention levels are usually quite low at 18 months follow-up measurements [133]. One way to solve this problem is to make the intervention more attractive to the participants by personalizing it. Personalized smartphone-based PA mHealth interventions may be more effective and preferred by participants over interventions with a generic program and advices or notifications [28,51]. Various researchers have suggested that personalization or tailoring of PA interventions will positively affect participants’ perception and engagement [59,134-136]. Some studies have attempted to personalize the intervention components manually and using automated approaches [137,138]. Manual automation (where the researcher inputs a large amount of collected data individually into every participant’s profile) has shown positive trends. Nevertheless, depending on the number of variables, the number of entries required for each participant, and the number of participants, this approach might be too time-consuming to become impractical [139,140]. More automated approaches, specifically machine learning or data mining, require minimum assistance during the utilization period, and are therefore promising for solving big data challenges, including behavior change interventions [113,194]. Rabbi et al [138,195] have already successfully implemented machine-learning solutions in various smartphone-based mHealth interventions, demonstrating their potential. However, machine learning science is in its early stage of development, and some questions still need to be answered and tested. One of them concerns the level of automation: Which one should the researcher choose for his or her particular intervention? Although full manual tracking is considered outdated and has a high data collection burden, fully automated tracking requires high data collection accuracy and may lower participants’ self-awareness; therefore, semiautomated tracking is currently the best solution [141,142].

As the articles listed in Table S10 of Multimedia Appendix 1 show, numerous technological advances are increasingly being used in smartphone-based PA mHealth interventions. One example concerns HR monitoring. Previously, HR monitoring PA interventions used separate devices. Currently, commercial activity trackers include built-in HR sensors, which can increase participants’ acceptance of the intervention. Another example concerns the use of a smartphone’s inbuilt GPS sensor, which can provide high accuracy of movement speed and location, among others; however, it is highly energy consuming and drains smartphone batteries very fast. The latest power management algorithms help to reduce the resource demands of continuous sensing, which ensures longer usability time, providing researchers with additional data collection opportunities [143].

### Implications for Future Research

On the basis of this review and in light of the widely used international reporting guidelines, several recommendations for future research can be inferred:

- Support uniformity of reporting by describing interventions and procedures in an adequate and consistent manner, using industry-recognized reporting guidelines, such as PRISMA, CONSORT, and SPIRIT [196].
- Develop and code interventions in a more systematic way, using recommended practices while taking into account new models that offer additional opportunities in behavior research [186]. Currently, the systematic approach is either not applied or various frameworks are being used (eg, different versions of taxonomy by Michie), which slows or even prevents knowledge transfer and evidence accumulation. After the first results will be yielded in the development of a methodology for linking BCTs to theoretical mechanisms and the Human Behavior-Change Project, more systematic solutions will become available [88,194].
- Meta-analyses, including modern mHealth solutions (eg, smartphones) and excluding outdated devices or methods (intervention based solely on SMS, PDAs, etc), provided there is a sufficient number of studies meeting the inclusion criteria.
- Profit from interdisciplinary collaboration while developing mHealth interventions. Various researchers and research groups working on the development of PA mHealth interventions have underlined the positive effect of collaboration between related stakeholders and experts in the domains of behavior change, software development, machine learning or data science, physiology, and public health [29,65,70,94,102,106,113,135,144]. A recent systematic review demonstrated that the collaboration of experts from various research domains greatly enhances the quality of the produced publications and research work in general [145].
- Perform more studies designed for adolescents, accounting for differences in levels of motivation and lifestyle compared with adults.
- Implement rapid study designs while evaluating the intervention (eg, MRT, Multiphase Optimization Strategy, Sequential Multiple Assignment Randomized Trial, etc) [32].
- Implement wearable activity monitors with built-in sensors (eg, HR and GPS) will provide more opportunities for data collection. Both commercial and research-grade trackers are advantageous. However, the collaboration of two domains, for instance ActiGraph and Garmin, is yet to bring fruitful results [197].
- Implement the latest findings of machine learning or data mining and artificial intelligence domains into behavior change interventions [88,138,194].
- Improve engagement with smartphone-based mHealth interventions by testing and implementing meaningful gamification and social networking features [120].
- Build the reward and engagement engine of the app in a way that users will become autonomously physically active over time and do not depend on an app, a tracker, or an intervention in perpetuity.

### Strengths and Limitations

The strength of this scoping review is the comprehensive search strategy, which allows the majority of published related articles to be included. Therefore, the scope of the review is wider than the scope of systematic reviews on smartphone-based mHealth interventions for PA promotion. However, a scoping review does not consider the methodological quality assessment of the included studies. Consequently, several studies had moderate methodological quality, which calls for their findings into question. It is important to emphasize that the included interventions developed and evaluated apps and activity trackers that provide sensor-based feedback on PA. Smartphone-based interventions related to chronic diseases other than cardiovascular diseases and obesity (eg, diabetes mellitus), preventive health issues (eg, alcohol abuse, smoking, and sports injuries), weight loss, diet, and nutrition were not included in this review. Finally, yet most importantly, only smartphone-based mHealth interventions were included in this review.

### Conclusions

Smartphone-based mHealth interventions aimed at PA promotion in adolescents and adults show promising results for effective behavior change. Although there is a plethora of published studies with adults, the number of studies and, consequently, the evidence base for adolescents is very limited. In the past few years, a growing number of researchers have developed multicomponent mHealth interventions that, in addition to the app, include commercial or research-grade activity trackers, which can provide additional insight into a participant’s lifestyle. Overall, the efficacy of smartphone-based mHealth PA interventions can be considerably improved through a more systematic approach to developing, reporting, and coding of the interventions. Specifically, researchers should aim to develop theory-based rather than theory-inspired interventions, which is currently challenging, as there is no consensus on development, evaluation, or coding practice. Finally, the current stage of behavior science advocates an interdisciplinary approach to the development of behavior change interventions, including innovative approaches such as machine learning and data mining.

## Appendix

Multimedia Appendix 1 Articles included in the scoping review.

## Abbreviations

BCT: behavior change technique
CALO-RE: Coventry, Aberdeen & London–Refined
COM-B: Capability, Opportunity, Motivation, Behaviour
CONSORT: Consolidated Standards of Reporting Trials
HE: healthy eating
HR: heart rate
MARS: Mobile App Rating Scale
mHealth: mobile health
MRT: microrandomized trial
MVPA: moderate-to-vigorous physical activity
PA: physical activity
PRISMA: Preferred Reporting Items for Systematic Reviews and Meta-Analyses
PRISMA-P: Preferred Reporting Items for Systematic Reviews and Meta-Analyses Protocols
RCT: randomized controlled trial
SB: sedentary behavior
SPIRIT: Standard Protocol Items: Recommendations for Interventional Trials
WHO: World Health Organization
