# Supplementary material for: Smartphone-Based Interventions for Physical Activity Promotion: Scoping Review of the Evidence Over the Last 10 Years
Source: JMIR Mhealth Uhealth. 2021 Jul 21;9(7):e24308. doi: 10.2196/24308 (PMC8339983; doi:10.2196/24308)
Supplement: Multimedia Appendix 1 [file mhealth_v9i7e24308_app1.docx]

# **Multimedia Appendix 1. Articles included in the scoping review.**

**Table S1.** Characteristics of the commercial smartphone application content analyses.

| **Author** | **Year** | **Target group** | **Targeted behavior** | **Evaluation framework/ Taxonomy used for coding** | **Number of apps included** | **App market name and category** | **Findings related to the theoretical background** |
| --- | --- | --- | --- | --- | --- | --- | --- |
| Cowan et al. [1] | 2012 | N/A | PA | Health behavior theory constructs: instrument developed by Doshi, Patrick, Sallis, and Calfas (2003) | 127 | Apple App Store: Health & Fitness category | Apps were generally observed to be lacking in theoretical content. Regression analyses indicated that higher priced apps and apps that addressed a broader activity spectrum were associated with higher total theory scores. |
| West et al. [2] | 2012 | N/A | Health and Fitness | The Health Education Curriculum Analysis Tool and the Precede-Proceed Model | 3336 | Apple App Store: Health & Fitness category | Apps related to healthy eating, physical activity, and personal health and wellness were more common than apps for substance abuse, mental and emotional health, violence prevention and safety, and sexual and reproductive health. Reinforcing apps were less common than predisposing and enabling apps. |
| Schoffman et al. [3] | 2013 | Children and adolescents <18 years | Obesity prevention, diet, PA | The Expert Committee for Pediatric Obesity Prevention's eight recommended strategies and seven behavioral targets | 57 | Apple App Store | Most apps reviewed lacked expert recommendations and could be strengthened by addition of comprehensive information about health behavior change and opportunities for goal setting. |
| Conroy et al. [4] | 2014 | N/A | PA | The CALO-RE (Coventry, Aberdeen & London – Refined) taxonomy (40 BCTs) | 67 | Apple App Store and Google Play: Health & Fitness category (top-ranked apps) | Most descriptions of apps incorporated fewer than **4 BCTs**. The most common techniques involved "providing instruction on how to perform exercises", "modeling how to perform exercises", "providing feedback on performance", "goal-setting for physical activity", and "planning social support/change". |
| Direito et al. [5] | 2014 | N/A | PA and Diet | The Taxonomy of Behaviour Change Techniques Used in Interventions (26 BCTs) | 40 | Apple App Store: Health & Fitness category (top-20 paid and top-20 free apps) | Apps included an average of **8.1 BCTs** (range 2-18), the number being slightly higher for paid (mean (M) = 9.7, range 2-18) than free apps (M = 6.6, range 3-14). The most frequently included BCTs were “provide instruction” (83% of the apps), “set graded tasks” (70%), and “prompt self-monitoring” (60%). Techniques such as “teach to use prompts/cues”, “agree on behavioral contract”, “relapse prevention” and “time management” were not present in the apps reviewed. |
| Middelweerd et al. [6] | 2014 | Adults | PA | The Taxonomy of Behaviour Change Techniques Used in Interventions (26 BCTs) | 64 | Apple iTunes and Google Play | On average, the reviewed apps included **5 BCTs** (range 2–8). Techniques such as "self-monitoring", 'providing feedback on performance", and "goal-setting" were used most frequently, whereas some techniques such as "motivational interviewing", "stress management", "relapse prevention", "self-talk", "role models", and "prompted barrier identification" were not. No differences in the number of behavior change techniques between free and paid apps, or between the app stores were found. |
| Yang et al. [7] | 2015 | N/A | PA | The behavior change technique taxonomy (v1) of 93 hierarchically clustered techniques | 100 | Apple iTunes and Google Play: Health & Fitness category (25 paid and 25 free apps from each marketplace) | Users identified an average of **6.6 BCTs** per app and most BCTs in the taxonomy were not represented in any apps. The most common BCTs involved "providing social support", "information about others’ approval", "instructions on how to perform a behavior", "demonstrations of the behavior", and "feedback on the behavior". A latent class analysis of BCT configurations revealed that apps focused on providing support and feedback as well as support and education. |
| Modave et al. [8] | 2015 | N/A | PA | American College of Sports Medicine guidelines | 30 | Apple App Store: Health & Fitness category (free apps) | A weighted scoring method based on the recommendations of the ACSM was developed to generate subscores for quality of programming content for aerobic (0-6 scale), resistance (0-6 scale), and flexibility (0-2 scale) components using the frequency, intensity, time, and type (FITT) principle. Only 3 apps scored above 50% on the aerobic component (M 0.7514, standard deviation (SD) 1.2150, maximum 4.1636), 4 scored above 50% on the resistance/strength component (M = 1.4525, SD = 1.2101, maximum 4.1094), and no app scored above 50% on the flexibility component (M = 0.1118, SD 0.2679, maximum 0.9816). Finally, only 1 app had an overall score (64.3%) above 50% (M = 2.3158, SD 1.911, maximum 9.0072). |
| Knight et al. [9] | 2015 | Adults | PA | Public health guidelines for physical activity (WHO/ Canadian physical activity guidelines for adults/ United States Department of Health and Human Services) | 379 | Apple iTunes and Google Play | Primary results demonstrated no apps (n=0) adhering to evidence-based guidelines for aerobic physical activity, and 7 out of 379 implementing evidence-based guidelines for resistance training physical activity. Technological features of apps included social networking (n=207), pairing with a peripheral health device (n=61), and measuring additional health parameters (n=139). Secondary results revealed 1 app that referenced physical activity guidelines (150 minutes/weekly of exercise), and demonstrated that apps were based on various physical activity reports (n=4) or personal expertise (n=2). |
| Schoeppe et al. [10] | 2017 | Children and adolescents <18 years | PA, SB and Diet | The Taxonomy of Behaviour Change Techniques Used in Interventions (26 BCTs);  Mobile App Rating Scale (MARS) | 25 | Apple iTunes and Google Play | Twenty-five apps were included targeting diet (n = 12), physical activity (n = 18) and sedentary behavior (n = 7). On a 5-point Mobile App Rating Scale, overall app quality was moderate (total score: 3.6). Functionality was the highest scoring domain (M = 4.1, SD: 0.6), followed by aesthetics (M= 3.8, SD: 0.8), and lower scoring for engagement (mean: 3.6, SD: 0.7) and information quality (M = 2.8, SD = 0.8). On average, **6 BCTs** were identified per app (range: 1–14); the most frequently used BCTs were "providing instructions" (n = 19), "general encouragement" (n = 18), "contingent rewards" (n = 17), and "feedback on performance" (n = 13). App quality ratings correlated positively with numbers of technical app features (rho = 0.42, p < 0.05) and BCTs included (rho = 0.54, p < 0.01). |
| Simões et al. [11] | 2018 | N/A | PA | The Taxonomy of Behaviour Change Techniques Used in Interventions (26 BCTs);  Mobile App Rating Scale (MARS) | 51 | Apple App Store, Google Play, Windows Phone Store | Of 51 apps included, none specified the age of the target group and only one mentioned the involvement of health professionals. Most apps offered the possibility to work in background (n=50) and allowed data sharing (n=40). Regarding physical activity, most apps measured steps and distance (n=11) or steps, distance, and time (n=17). Only 18 apps, all of which measured number of steps, followed the guidelines on recommendations for physical activity. On average, **5.5 BCTs** (SD 1.8) were identified per app; the most frequently used techniques were “provide feedback on performance” (n=50) and “prompt self-monitoring of behavior” (n=50). The overall quality score was 3.88 (SD 0.34). |

**Table S2.** Characteristics of the smartphone-based intervention review studies.

| **Author** | **Year** | **Target group** | **Targeted behavior** | **Taxonomy used for coding** | **Information on BCTs** | **Identified psychological theories** | **Number of studies included** | **Objective** | **Industry-recognized reporting guidelines** |
| --- | --- | --- | --- | --- | --- | --- | --- | --- | --- |
| Stephens et al. [12] | 2013 | N/A | PA and weight reduction | N/A | N/A | N/A | 7 | To determine user satisfaction and effectiveness of smartphone applications and text messaging interventions to promote weight reduction and PA. |  |
| Bort-Roig et al. [13] | 2014 | N/A | PA | N/A | N/A | Transtheoretical Model, Social Cognitive Theory | 26 | To systematically review evidence on smartphones and their viability for measuring and influencing PA. |  |
| Monroe et al. [14] | 2015 | N/A | PA | N/A | N/A | Social Cognitive Theory | 52 | To conduct a systematic review of mobile phone–based approaches for encouraging and assessing PA. |  |
| Payne et al. [15] | 2015 | N/A | Health behavior | N/A | N/A | Social Cognitive theory, Self-determination Theory | 24 | To systematically search and describe the literature on mobile apps used in health behavior interventions, describe the behavioral features and focus of health apps, and to evaluate the potential of apps to disseminate health behavior interventions. |  |
| Quelly et al. [16] | 2015 | Children and adolescents 9-19 years | Obesity combating | N/A | N/A | Theory of Planned Behavior, Social Cognitive Theory | 9 | To examine the impact of mobile app technology on obesity-related anthropometric, psychosocial, and behavioral outcomes in children and adolescents. | PRISMA |
| Coughlin et al. [17] | 2016 | N/A | PA | N/A | N/A | N/A | 15 | To review published studies on the acceptability and efficacy of smartphone apps designed to promote PA or to lose weight. |  |
| Direito et al. [18] | 2016 | Young people & adults | PA and SB | The behavior change technique taxonomy (v1) of 93 hierarchically clustered techniques | Frequently employed BCTs in intervention groups were “goal setting (behavior)” (81 % of the studies), “self-monitoring of behavior” (74 %), “social support (unspecified)” (65 %), “feedback on behavior” (55 %), “instruction on how to perform the behavior” (55 %), “adding objects to the environment” (48 %), “information about health consequences” (45 %) and “prompts/cues” (45 %). Other BCTs, such as “discrepancy between current behavior and goal” (0 %), “behavioral contract” (0 %), “behavioral experiments” (0 %), and “review of behavior goal(s)” (16 %), were never or seldom reported. | N/A | 21 | To compare the effectiveness of mHealth interventions to promote PA and reduce sedentary behavior in free-living young people and adults with a comparator exposed to usual care/minimal intervention; to determine whether, and to what extent, such interventions affect PA and sedentary behavior levels; to use the taxonomy of behavior change techniques to describe intervention characteristics | PRISMA |
| Dute et al. [19] | 2016 | Adolescents & adults 12-25 years | Healthy nutrition, PA and overweight prevention | The Taxonomy of Behaviour Change Techniques Used in Interventions (26 BCTs) | The techniques “prompt self-monitoring of behavior” and “provision of feedback on performance” are most often applied. Another frequently applied technique is “specific goal setting” and contingent rewards. | Transtheoretical model, Self-determination Theory | 15 | To explore how mobile apps can contribute to the promotion of healthy nutrition, PA, and prevention of overweight in adolescents and students |  |
| Matthews et al. [20] | 2016 | N/A | PA | N/A | N/A | Persuasive Systems Design model | 20 | To review the current state of mobile applications for a health behavioral change with an emphasis on applications that promote PA. The inbuilt persuasive features of these applications was evaluated using the Persuasive Systems Design model. |  |
| Schoeppe et al. [21] | 2016 | Children and adolescents (8-17 years) and adults (18-71 years) | PA, SB and Diet | N/A | Most of the interventions showing significant improvements in the behavioral and health outcomes included goal-setting, self-monitoring and performance feedback in the app design. Some efficacious interventions also incorporated other behavior change techniques, such as motivational messages, health education/tailored advice, reinforcement, gamification in the form of exergames, award and rewards, social support through interaction with peers and friendly team challenges. Moreover, there was no difference in the behavior change techniques incorporated in apps for children compared to those used in apps for adults. | Self-determination Theory, Transtheoretical Model, Social Cognitive Theory, Theory of Planned Behavior, Control Systems Theory of Self-regulation, the Behavior Change Wheel. | 27 | To systematically synthesize evidence for the efficacy of interventions that use apps to improve diet, PA and sedentary behavior for non-communicable disease prevention | PRISMA |
| Jee et al. [22] | 2017 | Adults | PA | N/A | N/A | N/A | 10 | To observe feasibility and applicability of mobile applications for PA intervention |  |
| Stuckey et al. [23] | 2017 | Adults | PA | N/A | Effective: feedback, motivational cuing, goal setting. Less or not effective: information and education, reminders, rewards or reinforcement, social support, gamification | Five A’s Model, Fogg Behavior Model, Learning Theory/Operant Conditioning, Self-Determination Theory, Social Cognitive Theory, Social Influence Theory, Theory of Reasoned Action, Transtheoretical Model (Stages of Change) | 18 | To determine whether smartphone-based interventions successfully encourage engagement in PA; to explore the success of interventions in different populations; to examine the key factors of interventions that successfully encouraged PA |  |
| Sullivan et al. [24] | 2017 | Adults | PA | N/A | Goal setting, self-monitoring, feedback, rewards, social support, and coaching seem to be especially helpful in increasing activity and healthy behaviors. Recommended techniques: identifying obstacles, restructuring negative attitudes, action planning, and modifying environmental factors. | Cognitive Behavior Therapy, Social Cognitive Theory | N/A | To review the evidence for fitness technology increasing PA in sedentary adults |  |

| **Table S3.** Characteristics of activity tracker content analyses. | | | | | | | | |  |
| --- | --- | --- | --- | --- | --- | --- | --- | --- | --- |
| **Author** | **Year** | **Target group** | **Targeted behavior** | **Evaluation criteria/ Taxonomy used for coding** | **Number of trackers included** | **Number of BCTs included (mean value)** | **BCTs present in all included devices** | **BCTs present in none of the included devices** |  |
| Duncan et al. [25] | 2017 | N/A | PA, SB, sleep | The CALO-RE taxonomy (40 BCTs) | 3 PA monitors | 25,6/40 | “providing information about others’ approval”, “providing normative information about others behavior”, “goal setting (behavioral and outcome)”, “goal review (behavioral and outcome)”, “prompt rewards contingent on progress toward goal”, “prompt rewards contingent on successful behavior”, “shaping”, “self-monitoring (behavior and outcome)”, “prompting focus on past success”, “providing feedback on performance”, “agreeing to behavioral contracts”, “facilitate social comparison”, “plan social support”, “prompt identification of role model”, “relapse prevention” | “model or demonstrate the behavior”, “prompt anticipated regret”, “prompt self-talk”, “fear arousal”, “prompt use of imagery”, “general communication skills training” |  |
| Mercer et al. [26] | 2016 | N/A | PA | The CALO-RE taxonomy (40 BCTs) | 7 PA monitors | 16.3/40 | “provide information about others’ approval”, “provide normative information about others’ behavior”, “prompt review of behavioral goals”, “provide rewards contingent on successful behavior”, “prompt self-monitoring of behavior”, “prompting focus on past success”, “provide feedback on performance”, “facilitate social comparison”, “plan social support/social change” | “barrier identification or problem-solving”, “set graded tasks”, “prompting generalization of a target behavior”, “environmental restructuring”, “agree behavioral contract”, “use of follow-up prompts”, “prompt identification as role model or position advocate”, “prompt anticipated regret”, “fear arousal”, “prompt self-talk”, “prompt use of imagery”, “relapse prevention or coping planning”, “stress management or emotional control training”, “motivational interviewing”, general communication skills training” |  |
| Lyons et al. [27] | 2014 | N/A | PA | The behavior change technique taxonomy (v1) of 93 hierarchically clustered techniques | 13 PA monitors | 9/40; 8/26 | N/A | N/A |  |

**Table S4.** Characteristics of activity tracker review studies.

| **Author** | **Year** | **Target group** | **Targeted behaviour** | **Evaluation criteria/ Taxonomy used for coding** | **Number of studies included** | **Industry-recognized reporting guidelines** |
| --- | --- | --- | --- | --- | --- | --- |
| Straiton et al. [28] | 2018 | Older, community-dwelling adults | PA | Validity and Reliability | 7 | PRISMA |
| de Vries et al. [29] | 2016 | Adults with Overweight or Obesity | PA | Effectiveness | 14\11 | PRISMA-P |
| Ridgers et al. [30] | 2016 | Children and adolescents (5-19) | PA | Feasibility and Effectiveness | 5 | PRISMA |
| Lewis et al. [31] | 2015 | Adults | PA | Efficacy and Feasibility | 11 | PRISMA |
| Evenson et al. [32] | 2015 | Adults and adolescents | PA, sleep | Validity, Reliability, Feasibility | 22 | PRISMA |
| Van Remoortel et al. [33] | 2012 | Healthy adults/ with chronic diseases | PA | Validity | 134 | - |

| **Table S5.** Characteristics of meta-analyses of PA intervention studies. | | | | | | |  |
| --- | --- | --- | --- | --- | --- | --- | --- |
| **Author** | **Year** | **Target group** | **Targeted behavior** | **Taxonomy used for coding** | **BCTs associated with more effective interventions** | **BCTs associated with less effective interventions** | **Industry-recognized reporting guidelines** |
| Michie et al. [34] | 2009 | Healthy adults | HE and PA | The Taxonomy of Behaviour Change Techniques Used in Interventions (26 BCTs) | “self-monitoring”, “intention formation”, “specific goal setting”, “review of behavioral goals”, “feedback on performance” | N/A |  |
| Williams et al. [35] | 2011 | Healthy adults | PA self-efficacy and behavior | The CALO-RE taxonomy (40 BCTs) | “provide information on consequences of the behavior in general”, “action planning”, “reinforcing effort or progress towards behavior”, “provide instruction”, “facilitate social comparison”, “time management”, “providing information on the consequences of performing physical activity”. | "set graded tasks", "use of follow up prompts", "relapse prevention" |  |
| Olander et al. [36] | 2013 | Obese adults | PA self-efficacy and behavior | The CALO-RE taxonomy (40 BCTs) | “prompt self-monitoring of behavioural outcome”, “plan social support/social change”, “teach to use prompts/cues”, “prompt practice”, “prompt rewards contingent on effort or progress towards behaviour” | ‘prompting generalization of a target behavior’ |  |
| Brannon et al. [37] | 2015 | Children (6-13 years) and adolescents (13-18 years) | PA and diet | The Taxonomy of Behaviour Change Techniques Used in Interventions (26 BCTs) | “providing consequences for behavior”, “providing information on other’s approval”, “prompting intention formation”, “self-monitoring”, “creating a behavioral contract” | "use of instruction" |  |
| O’Brien et al. [38] | 2015 | Adults 55-77 years | PA | The CALO-RE taxonomy (40 BCTs) | "feedback" | “information on where and when to perform the behavior”, “information on consequences of behavior to the individual” |  |
| Samdal et al. [39] | 2017 | Overweight and obese adults | HE and PA | The behavior change technique taxonomy (v1) of 93 hierarchically clustered techniques | Short- and long-term positive effects: “goal setting of behavior”, “self-monitoring of behavior”,  Short-term positive effects: “feedback on behavior”, “feedback on outcome of behavior”, “demonstration of the behavior”,  Long-term positive effects: “giving feedback on the outcome of behavior”, “setting graded task”, “adding objects to the environment” | "pros and cons of behavior change" | PRISMA |

**Table S6.** Characteristics of the smartphone-based intervention studies.

| **Pilot** | **Protocol** | **Author** | **Year** | **Target group** | **Sample size** | **Theoretical background** | **Study design** | **Study duration** | **Targeted behavior** | **Stand-alone or multi-component intervention** | **Principal outcome measures** | **Industry-recognized reporting guidelines** | **PA recommendations** |
| --- | --- | --- | --- | --- | --- | --- | --- | --- | --- | --- | --- | --- | --- |
| No | No | Toscos et al. [40] | 2008 | Adolescents 13 (female) | 8 | N/A | Field trial | 3 weeks | PA | Multi-component: application and pedometer | PA (daily step count) |  |  |
| No | No | Watterson et al. [41] | 2012 | Adolescents 11-15 | 140 | Self-Determination Theory, Basic Psychological Needs Theory | Dissertation: Patient-Centered Assessment and  Counseling for Exercise (PACE) survey, interview, 1 arm trial | 8 weeks | PA, Diet | Multi-component: application and pedometer | PA (minutes in MVPA), Diet (food logging) |  | Yes |
| No | No | Lu et al. [42] | 2013 | Adolescents 15-17 | 19 | N/A | 1 arm trial | 6 weeks | Fitness | Stand-alone: application | Fitness level (BMI) |  |  |
| No | No | Van Dantzig et al. [43] | 2013 | Adults 30-63 | 86 (experiment n=40; control n=46) | N/A | 2 arm trial | 6 weeks | SB | Stand-alone: application | SB (minutes) |  |  |
| No | Yes/No | Glynn et al. [44,45] | 2013/ 2014 | Adults 16+ | 78 (experiment n=37; control n=41) | Commercial app: Accupedo-Pro Pedometer app | 2 arm parallel RCT | 8 weeks | PA | Stand-alone: application | PA (daily step count) | CONSORT |  |
| No | No | Bond et al. [46] | 2014 | Adults 21-70 | 30 | N/A | 1 arm within-subjects experimental trial | 4 weeks | SB, PA | Multi-component: application and SenseWear Mini Armband activity monitor | SB (minutes) PA (minutes in MVPA) |  |  |
| No | No | Duncan et al. [47] | 2014 | Adults 35-54 | 301 (experiment n=205; control n=96) | Social Cognitive Theory, Self-Regulatory Theory | 2 arm RCT | 32 weeks | PA, Diet | Stand-alone: application | PA (questionnaire), Diet (questionnaire) | CONSORT |  |
| No | No | Lu et al. [48] | 2014 | Adolescents 14-15 | 35 (experiment n=20; control n=15) | N/A | 2 arm controlled trial + survey | 8 weeks | Fitness | Stand-alone: application | Fitness level (BMI) |  |  |
| No | Yes/No | Smith et al. [49,50] | 2014 | Adolescents 12-14 | 361 (experiment n=180; control n=181) | Self-Determination Theory and Social Cognitive Theory (specifically the trans-contextual model of motivation). | 2 arm RCT | 15 weeks | PA, SB | Multi-component: application and Actigraph GT3X accelerometer | Adiposity (BMI), PA intensity (counts/minute) , SB (ASAQ questionnaire), Muscular fitness (exercise), etc. | CONSORT | Yes |
| No | Yes/No | Recio-Rodríguez et al. [51,52] | 2014/ 2016 | Adults 70- | 633 (experiment n=315; control n=318) | N/A | 2 arm RCT | 12 weeks | PA, Diet | Multi-component: application and ActiGraph GT3X accelerometer | PA intensity (counts/minute) and MEDAS screener (diet) | CONSORT | Yes |
| No | No | Blackman et al. [53] | 2015 | Adolescents 12-13 | 27 | Fogg's behaviour model | 1 arm mixed-methods, quasi-experimental trial (no control group) | 6 weeks | PA | Stand-alone: application | PA (METs) |  |  |
| No | Yes/No | Direito et al. [54,55] | 2015 | Adolescents 14-17 | 51 (experiment I n=17; experiment II n=16; control n=18) | Commercial apps: Zombies, Run! and Get Running | 3 arm parallel RCT | 8 weeks | Cardiorespiratory fitness, PA | Stand-alone: application | Cardiorespiratory fitness (test), PA intensity (counts/minute) | CONSORT; SPIRIT |  |
| No | No | Garde et al. [56] | 2015 | Adolescents 8-13 | 47 (experiment n=26; control n=21) | Self-Determination Theory, Theory of Motivation in Videogames | 2 arm controlled trial | 2 weeks | PA | Multi-component: application and Tractivity activity monitor | PA (daily step count, minutes in MVPA) |  |  |
| Yes | No | Martin et al. [57] | 2015 | Adults 18-69 | 48 (experiment I n=16; (experiment II n=16; (experiment III n=16) | N/A | 3 arm sequential (2 phases) RCT pilot | 5 weeks | PA | Multi-component: application and Fitbug Orb activity monitor | PA (daily step count, minutes in MVPA) | CONSORT |  |
| Yes | No | Rabbi et al. [58] | 2015 | Adults 18-49 | 17 (experiment n=9; control n=8) | Learning Theory, Social Cognitive Theory, and the Fogg Behavior Model | 2 arm RCT | 3 weeks | PA, Diet | Stand-alone: application | PA (minutes), SB (minutes) and Diet (caloric intake) | CONSORT | Yes |
| No | Yes/No | Pellegrini et al. [59]; Spring et al. [60] | 2015/ 2018 | Adults 18-65 | 212 (experiment I n=84; (experiment II n=84, control n=44) | The Synergy Hypothesis, grounded in goal systems theory | 3 arm RCT | 12 weeks | Diet, PA, SB | Multi-component: application and the Shimmer accelerometer | Diet, PA, SB (measured in a composite score by an app) | CONSORT |  |
| Yes | No | Choi et al. [61] | 2016 | Adults 18-40 | 30 (experiment n=15; control n=15) | Social Cognitive Theory | 2 arm RCT | 3 weeks | PA | Multi-component: application and Fitbit Ultra activity monitor | PA (daily step count) |  |  |
| No | Yes | Duncan et al. [62] | 2016 | Adults 18-55 | 64 (experiment n=32; control n=32) | Social Cognitive Theory, Self-Regulatory Theory | 2 arm RCT | 9 weeks | PA, SB, sleep | Multi-component: application and Fitbit Charge HR activity monitor or GENEActiv accelerometer | PA (minutes in MVPA) , sleep (minutes) |  |  |
| No | No | Harries et al. [63] | 2016 | Adults 22-40 (male) | 152 (experiment I n=53; experiment II n=50; control n=49) | N/A | 3 arm RCT | 8 weeks | PA | Stand-alone: application | PA (daily step count) |  |  |
| No | No | King et al. [64] | 2016 | Adults 45-81 | 89 (experiment I n=22; experiment II n=22; experiment III n=21; Control n=24) | Social Cognitive theory and self-regulatory principles of behavior change, social influence perspectives, reinforcement scheduling, attachment and nurturance motives, as well as gamification | 4 arm parallel RCT | 8 weeks | PA, SB | Stand-alone: application | PA (minutes in MVPA) and SB (minutes) | CONSORT |  |
| No | Yes | Lubans et al. [65] | 2016 | Adolescents 12-14 | 640 | Self-Determination Theory and Social Cognitive Theory (specifically the trans-contextual model of motivation). | 2 arm cluster RCT | 10 weeks | PA, SB, sleep | Multi-component: application and GENEActiv accelerometer | Muscular fitness (exercise), PA (minutes in MVPA), etc. | CONSORT; SPIRIT | Yes |
| No | No | Lubans et al. [66] | 2016 | Adolescents 12-14 | 361 (experiment n=180; control n=181) | Self-Determination Theory and Social Cognitive Theory (specifically the trans-contextual model of motivation). | Quantitative follow-up: Adiposity (BMI), PA intensity (counts/minute) , SB (ASAQ questionnaire), Muscular fitness (exercise), etc. | N/A | PA, SB | Multi-component: application and Actigraph GT3X accelerometer | Adiposity (BMI), PA intensity (counts/minute) , SB (ASAQ questionnaire), Muscular fitness (exercise), etc. | CONSORT | Yes |
| No | No | Rospo et al. [67] | 2016 | Adults 20-55 | 33 (experiment I n=8; (experiment II n=12, experiment III n=13) | N/A | 3 arm RCT  +follow up interview | 2 weeks | Cardiorespiratory fitness | Multi-component: application, optical heart rate monitoring module, pedometer | Weight (kg), Blood Pressure, PA (daily step count), Heart rate (beats per minute) |  |  |
| Yes | No | Voth et al. [68] | 2016 | Adults 19-70 | 52 (experiment n=23; control n=19) | Social Cognitive Theory | 2 arm randomized experimental pilot study | 8 weeks | PA | Stand-alone: application | Engagement in exercise bouts, frequency of self-monitoring (surveys and questionnaires) |  |  |
| Yes | No | Walsh et al. [69] | 2016 | Adults 17-26 | 55 (experiment n=28; control n=27) | Commercial app: Accupedo-Pro Pedometer app + The COM-B model and the Behavior Change Wheel | 2 arm RCT Pilot | 5 weeks | PA | Stand-alone: application | PA (daily step count) |  | Yes |
| Yes | Yes/No | Shin et al. [70,71] | 2016/ 2017 | Adults 19-45 | 98 (experiment I n=32; (experiment II n=34; control n=32) | N/A | 3 arm RCT | 12 weeks | PA, Obesity | Multi-component: application + Fitmeter accelerometer | Weight loss (% of the baseline measurement) and PA (METs) |  |  |
| No | No | Fanning et al. [72] | 2017 | Adults 30-54 | 116 (experiment I n=31; (experiment II n=26, experiment III n=30, control n=29) | Social Cognitive Theory | 4 arm factorial RCT | 12 weeks | PA | Multi-component: application and Actigraph GT1M accelerometer | PA intensity (counts/minute) | CONSORT | Yes |
| No | No | Gaudet et al. [73] | 2017 | Adolescents 13-14 | 46 (experiment n=23; control n=23) | The transtheoretical model of health behavior change | 2 arm quasi-experimental crossover design | 7 weeks | PA | Stand-alone: actical accelerometer Philips and Fitbit Charge HR activity monitor | Heart rate (beats per minute), daily step count, PA (minutes in MVPA), SB (minutes) |  |  |
| No | No | Klasnja et al. [74] | 2018 | Adults 18-60 | 37 | N/A | MRT | 6 weeks | PA | Multi-component: application and Jawbone Up Move activity monitor | PA (daily step count) |  |  |
| No | No | Korinek et al. [75] | 2018 | Adults 40–65 | 20 | Social Cognitive Theory | 1 arm trial + exit interviews/surveys | 14 weeks | PA | Multi-component: application and Fitbit Zip activity monitor | PA (daily step count) |  |  |
| No | Yes | Recio-Rodriguez et al. [76] | 2018 | Adults 20-65 | 700 | N/A | 2 arm RCT | 12 weeks | PA, SB, Diet | Multi-component: application, ActiGraph GT3X accelerometer and ActivPal activity monitor | Weight loss (kg), PA intensity (counts/minute and IPAQ questionnaire), sitting time (min/wk), caloric intake (kcal/d). | SPIRIT | Yes |
| No | No | Van Woudenberg et al. [77] | 2018 | Adolescents 11-14 | 190 (experiment n=93; control n=97) | Self-Determination Theory | 2 arm clustered RCT | 2 weeks | PA | Multi-component: application and Fitbit Flrex activity monitor | PA (daily step count) |  |  |

**Table S7.** Characteristics of the qualitative formative studies.

| **Author** | **Year** | **Target group** | **Sample size** | **Target behavior** | **Method of data collection** |
| --- | --- | --- | --- | --- | --- |
| Rabin et al. [78] | 2011 | Adults (23-60) | 14 | Physical Activity | Survey and semi-structured interview |
| Dennison et al. [79] | 2013 | Adults (18-50) | 19 | Health Behavior Change | Focus group discussion |
| Ehlers et al. [80] | 2014 | Adults (30-64) | 120 | Physical Activity | Online survey |
| Gowin et al. [81] | 2015 | Adults (18-30) | 27 | Health and Fitness | Interview |
| Miyamoto et al. [82] | 2016 | Adults (25-64) | 30 | Health Behavior Change | Focus group discussion |
| Arteaga et al. [83] | 2010 | Adolescents 12-17 | 5 | Physical Activity | Center for Disease Control and Prevention (CDC) survey; focus group discussion; interview |

**Table S8.** Characteristics of the application development descriptive studies.

| **Author** | **Year** | **Target group** | **Sample size** | **Theoretical background** | **Target behavior** | **Common reported methods and practices for systematic development, evaluation and reporting** |
| --- | --- | --- | --- | --- | --- | --- |
| Ayubi et al. [84] | 2012 | N/A | N/A | Health Belief Model, Theory of Planned Behavior, Social Cognitive Theory, Self-Efficacy, Technology Acceptance Model, and Fogg Behavioral Model | PA |  |
| Hebden et al. [85] | 2012 | Adults 18-35 | 10 | N/A | PA |  |
| King et al. [86] | 2013 | Adults 45-81 | 68 | Social Cognitive theory and self-regulatory principles of behavior change, social influence perspectives, reinforcement scheduling, attachment and nurturance motives, as well as gamification | PA, SB | Physical activity recommendations |
| Lubans et al. [87] | 2014 | Adolescents 12-14 | 42 (male) | Self-Determination Theory and Social Cognitive Theory (specifically the trans-contextual model of motivation). | PA, SB |  |
| Alnasser et al. [88] | 2016 | Adults 18+ | 10 | Social Cognitive Theory | Weight loss | BCTs taxonomies |
| Vos et al. [89] | 2016 | Adults | 28 | N/A | PA | MARS |
| Middelweerd et al. [90] | 2018 | Adults 18-30 | N/A | Social cognitive theory | PA | BCTs taxonomies |

**Table S9.** Characteristics of the qualitative follow-up studies.

| **Author** | **Year** | **Target group** | **Sample size** | **Theoretical background** | **Target behavior** | **Method of data collection** |
| --- | --- | --- | --- | --- | --- | --- |
| Chang et al. [91] | 2012 | Adults | 68 | N/A | Well-Being | Online survey |
| Middelweerd et al. [92] | 2015 | Adults (18-23) | 30 | N/A | PA | Focus group discussion |
| Arteaga et al. [93] | 2012 | Adolescents 15-18 | 51 | N/A | PA | Survey; Interview |
| Arteaga et al. [94] | 2012 | Adolescents 13-15 | 5 | Theory of Meaning Behavior, Theory of Planned Behavior, 5 Factor Model of Personality | PA | Interview |
| Lu et al. [95] | 2013 | Adolescents 15-17 | 12 | N/A | Fitness | Pre-study and post-study questionnaire |
| Casey [96] | 2014 | Adults 17-62 | 12 | N/A | PA | Semi-structured interviews |
| Herrmann et al. [97] | 2017 | Adults 18+ | 47 | Theory of Planned Behavior, The Functional Triad | PA | Survey |

**Table S10**. Characteristics of the related articles.

| **Keyword** | **Author** | **Year** | **Title** | **Type of study/ methodology** | **Objective** |
| --- | --- | --- | --- | --- | --- |
| BCT | Abraham et al. [98] | 2008 | A Taxonomy of Behavior Change Techniques Used in Interventions | Taxonomy development | The objective of this study is to develop a Taxonomy of Behavior Change Techniques (26 BCTs with definitions) |
| GPS | Wiehe et al. [99] | 2008 | Using GPS-enabled cell phones to track the travel patterns of adolescents | Trial | Overall objective was to test the feasibility of using GPS-enabled cell phones to track an adolescent's travel patterns and gather daily diary data |
| GPS | Wiehe et al. [100] | 2008 | Adolescent Travel Patterns: Pilot Data Indicating Distance from Home Varies by Time of Day and Day of Week | Trial | Pilot feasibility study was performed to demonstrate the use of this technology in studying adolescent health-related behaviors and to demonstrate patterns and variability of activity by time of day and day of week. |
| Profiling | Kukafka et al. [101] | 2009 | An Evidence-based Decision Aid to Help Patients Set Priorities for Selecting Among Multiple Health Behaviors | Interviews | To explore how patients and providers respond to Tailored Lifestyle Conversations (TLC), an evidence-based decision aid to help patients set priorities for selecting among multiple health behavior change goals, researchers conducted a study utilizing key informant interviews. |
| GPS | Lu et al. [102] | 2010 | The Jigsaw Continuous Sensing Engine for Mobile Phone Applications | Narrative article | We present the design, implementation and evaluation of the Jigsaw continuous sensing engine, which balances the performance needs of the application and the resource demands of continuous sensing on the phone. |
| BCT | Michie et al. [103] | 2011 | A refined taxonomy of behaviour change techniques to help people change their physical activity and healthy eating behaviours: the CALO-RE taxonomy | Taxonomy development | This study aimed to extend the scope and improve the reliability of a 26-item taxonomy of behaviour change techniques developed by Abraham and Michie into A refined CALORE taxonomy (40 BCTs with definitions). |
| BCT | Michie et al. [104] | 2011 | The behaviour change wheel: A new method for characterising and designing behaviour change interventions | Framework development | This paper evaluates existing frameworks of behaviour change interventions, and develops and evaluates a new framework aimed at overcoming limitations of previous frameworks |
| Behavior Change Theory | Riley et al. [105] | 2011 | Health behavior models in the age of mobile interventions: are our theories up to the task? | Methodological Review | The study aims to determine how health behavior theories are applied to mobile interventions. |
| mHealth/apps | Klasnja et al. [106] | 2012 | Healthcare in the pocket: Mapping the space of mobile-phone health interventions | Methodological Review | This paper provides an overview of this rapidly growing body of work. Researchers describe the features of mobile phones that make them a particularly promising platform for health interventions, and identify five basic intervention strategies that have been used in mobile-phone health applications across different health conditions. Finally, researchers outline the directions for future research that could increase our understanding of functional and design requirements for the development of highly effective mobile-phone health interventions. |
| Profiling | Li et al. [107] | 2012 | Using Context to Reveal Factors that Affect Physical Activity | Trial | We explored the use of contextual information, such as events, places, and people, to support reflection on the factors that affect physical activity. |
| RCT alternatives for mHealth | Nilsen et al. [108] | 2012 | Advancing the Science of mHealth | Workshop report | mHealth requires a solid, interdisciplinary scientific approach that pairs the rapid change associated with technological progress with a rigorous evaluation approach. The mHealth Evidence Workshop and the NIH mHealth Training Institutes were both designed to address and further develop this scientific approach to mHealth. |
| BCT | Michie et al. [109] | 2013 | The Behavior Change Technique Taxonomy (v1) of 93 Hierarchically Clustered Techniques: Building an International Consensus for the Reporting of Behavior Change Interventions | Taxonomy development | The objective of this study is to develop an extensive, consensually agreed hierarchically structured taxonomy of techniques [behavior change techniques (BCTs)] used in behavior change interventions. |
| Behavior Change Theory | Spring et al. [110] | 2013 | Healthy Apps: Mobile Devices for Continuous Monitoring and Intervention | Methodological Review | As new technical capabilities to observe behavior continuously in context make it possible to tailor interventions in real time, the way we understand and try to influence behavior will change fundamentally. This article aims to shed a light on the developments in this domain. |
| mHealth/apps | Turner-McGrievy et al. [111] | 2013 | Comparison of traditional versus mobile app self-monitoring of physical activity and dietary intake among overweight adults participating in an mHealth weight loss program | Trial | The purpose of this study was to assess the relationship between diet (mobile app, website, or paper journal) and PA (mobile app vs no mobile app) self-monitoring and dietary and PA behaviors. |
| RCT alternatives for mHealth | Kumar et al. [112] | 2013 | Mobile Health Technology Evaluation: The mHealth Evidence Workshop | Workshop report | The current paper presents the results of the workshop. |
| Activity tracking | Hirsch et al. [113] | 2014 | Using MapMyFitness to place physical activity into neighborhood context | Narrative article | An overview of MapMyFitness, including data tracked, user information, and geographic scope, is explored. |
| BCT | Dusseldorp et al. [114] | 2014 | Combinations of Techniques That Effectively Change Health Behavior: Evidence From Meta-CART Analysis | Classification and regression trees | The aim was to identify particular combinations of BCTs that explain intervention success. |
| Activity tracking | Case et al. [115] | 2015 | Accuracy of Smartphone Applications and Wearable Devices for Tracking Physical Activity Data | Trial | The objective of this study was to evaluate the accuracy of smartphone applications and wearable devices compared with direct observation of step counts, a metric successfully used in interventions to improve clinical outcomes. |
| BCT | Belmon et al. [116] | 2015 | Dutch Young Adults Ratings of Behavior Change Techniques Applied in Mobile Phone Apps to Promote Physical Activity: A Cross-Sectional Survey | Cross-sectional online survey | This study aimed to explore young adults’ opinions regarding BCTs (including self-regulation techniques) applied in mobile phone physical activity apps, and to examine associations between personality characteristics and ratings of BCTs applied in physical activity apps. |
| BCT | Kok et al. [117] | 2015 | A taxonomy of behaviour change methods: an Intervention Mapping approach | Taxonomy presentation | In this paper, researchers introduce the Intervention Mapping (IM) taxonomy of behaviour change methods and its potential to be developed into a coding taxonomy. |
| BCT | Michie et al. [118] | 2015 | Behaviour change techniques: the development and evaluation of a taxonomic method for reporting and describing behaviour change interventions (a suite of five studies involving consensus methods, randomised controlled trials and analysis of qualitative data) | Report | The Behavior Change Technique Taxonomy (v1) (93 BCTs with definitions) |
| Behavior Change Theory | Hardcastle et al. [119] | 2015 | Motivating the unmotivated: how can health behavior be changed in those unwilling to change? | Narrative article | We offer some theory- derived suggestions on how to engage unmotivated individuals to increase their participation in health-promoting behaviors. |
| GPS | Benson et al. [120] | 2015 | Reliability and validity of a GPS-enabled iPhone “app” to measure physical activity | Trial | This study assessed the validity and reliability of an iPhone “app” and two sport-specific global positioning system (GPS) units to monitor distance, intensity and contextual physical activity. |
| mHealth/apps | Neubeck et al. [121] | 2015 | The mobile revolution—using smartphone apps to prevent cardiovascular disease | Methodological Review | In this Review, we assess the current literature and content of existing apps that target patients with CVD risk factors and that can facilitate behaviour change. We present an overview of the current literature on mobile technology as it relates to prevention and management of CVD. We also evaluate how apps can be used throughout all age groups with different CVD prevention needs. |
| mHealth/apps | Aitken et al. [122] | 2015 | Patient Adoption of mHealth | Report | This report provides an update to the analysis of the mHealth app landscape published by the IMS Institute for Healthcare Informatics in 2013. The primary focus of this report is on the consumer or patient use of mHealth apps. The mHealth app availability and usage information is focused on apps available to the general public that support everyday healthcare management in conjunction with their healthcare provider. While the information is agnostic to geographic boundaries, this report is primarily focused on the U.S. healthcare system. |
| mHealth/apps | Dallinga et al. [123] | 2015 | App use, physical activity and healthy lifestyle: a cross sectional study | A cross sectional online survey | The aim of this study was to determine the relationship between the use of apps and changes in physical activity, health and lifestyle behaviour, and self-image of short and long distance runners. |
| mHealth/apps | Gillman et al. [124] | 2015 | Effects of Performance Versus Game-Based Mobile Applications on Response to Exercise | Trial | This study tested a performance feedback-based app compared to a game-based app to examine their effects on aspects of immediate response to an exercise bout. |
| mHealth/apps | Stoyanov et al. [125] | 2015 | Mobile App Rating Scale: A New Tool for Assessing the Quality of Health Mobile Apps | Development of the quality assessment tool | The objective of this study was to develop a reliable, multidimensional measure for trialling, classifying, and rating the quality of mobile health apps. |
| mHealth/apps | VonHoltz et al. [126] | 2015 | Use of Mobile Apps: A Patient-centered Approach | Survey | This study explored what smartphone health applications (apps) are used by patients, how they learn about health apps, and how information about health apps is shared. |
| PA | Thompson et al. [127] | 2015 | Multidimensional Physical Activity: An Opportunity, Not a Problem | Narrative article | It is hypothesized that it will be important to improve the way in which these data are used and portrayed to provide a more accurate and integrated picture of an individual’s physical activity that cuts across the biologically important dimensions as well as using this information to offer people a smorgasbord of physical activity options and choices. |
| Profiling | Kukafka et al. [128] | 2015 | Optimizing Decision Support for Tailored Health Behavior Change Applications | Questionnaires | The study was to identify primary determinants of health behavior choices made by patients after using the system. Using discriminant analysis an optimal set of predictors was identified. |
| BCT | Michie et al. [129] | 2016 | From Theory-Inspired to Theory-Based Interventions: A Protocol for Developing and Testing a Methodology for Linking Behaviour Change Techniques to Theoretical Mechanisms of Action | Study Protocol | This research aims to develop and test a methodology for linking BCTs to their mechanisms of action. |
| mHealth/apps | Edwards et al. [130] | 2016 | Gamification for health promotion: systematic review of behaviour change techniques in smartphone apps | Coding of apps | Smartphone games that aim to alter health behaviours are common, but there is uncertainty about how to achieve this. We systematically reviewed health apps containing gaming elements analysing their embedded behaviour change techniques. |
| mHealth/apps | Harari et al. [131] | 2016 | Using Smartphones to Collect Behavioral Data in Psychological Science: Opportunities, Practical Considerations, and Challenges | Narrative article | In this article, we have drawn on the lessons from the first wave of smartphone-sensing research to highlight areas of opportunity for psychological research, present practical considerations for designing smartphone studies, and discuss the ongoing methodological and ethical challenges associated with research in this domain. |
| mHealth/apps | Lobelo et al. [132] | 2016 | The Wild Wild West: A Framework to Integrate mHealth Software Applications and Wearables to Support Physical Activity Assessment, Counseling and Interventions for Cardiovascular Disease Risk Reduction | Methodological Review | This paper reviews the validity, utility and feasibility of implementing mHealth technology in clinical settings and proposes an organizational framework to support PA assessment, counseling and referrals to community resources for CVD risk reduction interventions. |
| mHealth/apps | Recio et al. [133] | 2016 | The role of interdisciplinary research team in the impact of health apps in health and computer science publications: a systematic review | Methodological Review | This study aims to identify, study and evaluate the role of interdisciplinary research teams in the development of articles and applications in the field of mHealth. |
| mHealth/apps | Stoyanov et al.[134] | 2016 | Development and Validation of the User Version of the Mobile Application Rating Scale (uMARS) | Development of the quality assessment tool | This study describes the development and reliability testing of an end-user version of the MARS (uMARS). |
| RCT alternatives for mHealth | Pham et al. [135] | 2016 | Beyond the Randomized Controlled Trial: A Review of Alternatives in mHealth Clinical Trial Methods | Methodological Review | Given the recent development of alternative evaluation methodologies and tools to automate mHealth research, we sought to determine the breadth of these methods and the extent that they were being used in clinical trials. |
| RCT alternatives for mHealth | White et al. [136] | 2016 | Designing evaluation plans for health promotion mHealth interventions: a case study of the Milk Man mobile app | Methodological Review | This paper illustrates an evaluation planning approach for mHealth interventions that could be adapted for use by health promotion practitioners and researchers. |
| Activity tracking | Lyons et al. [137] | 2017 | Motivational Dynamics Of Wearable Activity Monitors | Narrative article | Researchers discuss the rationale for using specific types of wearables and recommendations for their implementation, which can be used by health and fitness professionals to evaluate various wearable devices (and/or apps). |
| Activity tracking | Wright et al. [138] | 2017 | How consumer physical activity monitors could transform human physiology research | Methodological Review | This review focuses on the unprecedented opportunities that consumer physical activity monitors offer for human physiology and pathophysiology research because of their ability to measure activity continuously under real-life conditions and because they are already widely used by consumers. |
| Automation | Choe et al. [139] | 2017 | Semi-Automated Tracking: A Balanced Approach for Self-Monitoring Applications | Narrative article | In the following, we define and characterize semi-automated tracking, examine three related design considerations, and provide examples of semiautomated tracking applications in the domains of sleep, mood, and food tracking to demonstrate strategies we developed. |
| BCT | Michie et al. [140] | 2017 | Developing and Evaluating Digital Interventions to Promote Behavior Change in Health and Health Care: Recommendations Resulting From an International Workshop | Workshop report | This article discusses these challenges and provides recommendations aimed at accelerating the rate of progress in digital behavior intervention research and practice. |
| mHealth/apps | Carroll et al. [141] | 2017 | Who Uses Mobile Phone Health Apps and Does Use Matter? A Secondary Data Analytics Approach | Survey | The objectives of our study were to (1) to describe the sociodemographic characteristics associated with health app use in a recent US nationally representative sample; (2) to assess the attitudinal and behavioral predictors of the use of health apps for health promotion; and (3) to examine the association between the use of health-related apps and meeting the recommended guidelines for fruit and vegetable intake and physical activity. |
| mHealth/apps | Ernsting et al. [142] | 2017 | Using Smartphones and Health Apps to Change and Manage Health Behaviors: A Population-Based Survey | Survey | The aim of this study was to explore (1) the extent of smartphone and health app use, (2) sociodemographic, medical, and behavioral correlates of smartphone and health app use, and (3) associations of the use of apps and app characteristics with actual health behaviors. |
| mHealth/apps | Hoj et al. [143] | 2017 | How Do Apps Work? An Analysis of Physical Activity App Users’ Perceptions of Behavior Change Mechanisms | A cross sectional online survey | The purpose of this study was to identify the mechanisms by which the use of physical activity apps may influence the users’ physical activity behavior. |
| Profiling | Pirolli et al. [144] | 2017 | Implementation Intention and Reminder Effects on Behavior Change in a Mobile Health System: A Predictive Cognitive Model | Trial | The aim of this experiment was to manipulate the effects of implementation intentions on daily behavioral goal success in ways predicted by the ACT-R theory concerning mHealth reminder scheduling. |
| RCT alternatives for mHealth | Moller et al. [145] | 2017 | Applying and advancing behavior change theories and techniques in the context of a digital health revolution: Proposals for more effectively realizing untapped potential | Narrative article | This paper offers proposals for better leveraging the potential advantages of digital health platforms, and reviews three of the cutting edge methods for doing so: optimization designs, dynamic systems modeling, and social network analysis. |
| JITAI | Schembre et al. [146] | 2018 | Just-in-Time Feedback in Diet and Physical Activity Interventions: Systematic Review and Practical Design Framework | Systematic Review | The goals of this systematic review were to summarize data on the content characteristics of feedback messaging used in diet and physical activity (PA) interventions and to develop a practical framework for designing just-in-time feedback for behavioral interventions. |
| mHealth/apps | Feldman et al. [147] | 2018 | Harnessing mHealth technologies to increase physical activity and prevent cardiovascular disease | Methodological Review | Researchers hope to provide a thorough overview of the mHealth landscape, in addition to highlighting many of the administrative, reimbursement and patient privacy challenges of using these technologies in patient care. Finally, we propose a behavior change model and checklist for clinicians to assist patients utilize mHealth technology to best achieve meaningful changes in daily physical activity levels. |
| mHealth/apps | McCallum et al. [148] | 2018 | Evaluating the Impact of Physical Activity Apps and Wearables: Interdisciplinary Review | Scoping review | This study aimed to (1) explore the extent to which evaluations of physical activity apps and wearables: employ rapid research designs; assess engagement, acceptability, as well as effectiveness; use efficient data collection methods; and (2) describe which dimensions of engagement and acceptability are assessed. |

## **REFERENCES**

1. Cowan LT, van Wagenen SA, Brown BA, Hedin RJ, Seino-Stephan Y, Hall PC, et al. Apps of Steel: Are Exercise Apps Providing Consumers With Realistic Expectations?: A Content Analysis of Exercise Apps for Presence of Behavior Change Theory. Heal Educ Behav. 2012;40(2):133–139. PMID: 22991048

2. West JH, Hall PC, Hanson CL, Barnes MD, Giraud-Carrier C, Barrett J. There’s an app for that: Content analysis of paid health and fitness apps. J Med Internet Res. 2012;14(3):1–12. PMID: 22584372

3. Schoffman DE, Turner-McGrievy G, Jones SJ, Wilcox S. Mobile apps for pediatric obesity prevention and treatment, healthy eating, and physical activity promotion: Just fun and games? Transl Behav Med. 2013;3(3):320–325. PMID: 24073184

4. Conroy DE, Yang CH, Maher JP. Behavior change techniques in top-ranked mobile apps for physical activity. Am J Prev Med. Elsevier; 2014;46(6):649–652. PMID: 24842742

5. Direito A, Pfaeffli Dale L, Shields E, Dobson R, Whittaker R, Maddison R. Do physical activity and dietary smartphone applications incorporate evidence-based behaviour change techniques? BMC Public Health. 2014;14(1):1–7. PMID: 24965805

6. Middelweerd A, Mollee JS, van der Wal CN, Brug J, te Velde SJ. Apps to promote physical activity among adults: A review and content analysis. Int J Behav Nutr Phys Act. 2014;11(1):1–9. PMID: 25059981

7. Yang CH, Maher JP, Conroy DE. Implementation of behavior change techniques in mobile applications for physical activity. Am J Prev Med. Elsevier; 2015;48(4):452–455. PMID: 25576494

8. Modave F, Bian J, Leavitt T, Bromwell J, Harris III C, Vincent H. Low Quality of Free Coaching Apps With Respect to the American College of Sports Medicine Guidelines: A Review of Current Mobile Apps. JMIR mHealth uHealth. 2015;3(3):e77. PMID: 26209109

9. Knight E, Stuckey MI, Prapavessis H, Petrella RJ. Public Health Guidelines for Physical Activity: Is There an App for That? A Review of Android and Apple App Stores. JMIR mHealth uHealth. 2015;3(2):e43. PMID: 25998158

10. Schoeppe S, Alley S, Rebar AL, Hayman M, Bray NA, Van Lippevelde W, et al. Apps to improve diet, physical activity and sedentary behaviour in children and adolescents: A review of quality, features and behaviour change techniques. Int J Behav Nutr Phys Act. International Journal of Behavioral Nutrition and Physical Activity; 2017;14(1):1–10. PMID: 28646889

11. Simões P, Amaral J, Rodrigues M, Rocha NP, Silva AG, Queirós A. Features, Behavioral Change Techniques, and Quality of the Most Popular Mobile Apps to Measure Physical Activity: Systematic Search in App Stores. JMIR mHealth uHealth. 2018;6(10):e11281. PMID: 30368438

12. Stephens J, Allen J. Mobile Phone Interventions to Increase Physical Activity and Reduce Weight. J Cardiovasc Nurs. 2013;28(4):320–329. PMID: 22635061

13. Bort-Roig J, Gilson ND, Puig-Ribera A, Contreras RS, Trost SG. Measuring and influencing physical activity with smartphone technology: A systematic review. Sport Med. 2014;44(5):671–686. PMID: 24497157

14. Monroe CM, Thompson DL, Bassett DR, Fitzhugh EC, Raynor HA. Usability of Mobile Phones in Physical Activity–Related Research: A Systematic Review. Am J Heal Educ. 2015;46(4):196–206. PMID: 103686931

15. Payne HE, Lister C, West JH, Bernhardt JM. Behavioral Functionality of Mobile Apps in Health Interventions: A Systematic Review of the Literature. JMIR mHealth uHealth. 2015;3(1):e20. PMID: 25803705

16. Quelly SB, Norris AE, Dipietro JL. Impact of mobile apps to combat obesity in children and adolescents: A systematic literature review. J Spec Pediatr Nurs. 2015;21(1):5–17. PMID: 26494019

17. Coughlin SS, Whitehead M, Sheats JQ, Mastromonico J, Smith S. A Review of Smartphone Applications for Promoting Physical Activity. Jacobs J community Med. 2016;2(1). PMID: 27034992

18. Direito A, Maddison R, Rawstorn J, Whittaker R, Carraça E. mHealth Technologies to Influence Physical Activity and Sedentary Behaviors: Behavior Change Techniques, Systematic Review and Meta-Analysis of Randomized Controlled Trials. Ann Behav Med. Annals of Behavioral Medicine; 2016;51(2):226–239. PMID: 27757789

19. Dute DJ, Bemelmans WJE, Breda J. Using Mobile Apps to Promote a Healthy Lifestyle Among Adolescents and Students: A Review of the Theoretical Basis and Lessons Learned. JMIR mHealth uHealth. 2016;4(2):e39. PMID: 27150850

20. Matthews J, Win KT, Oinas-Kukkonen H, Freeman M. Persuasive Technology in Mobile Applications Promoting Physical Activity: a Systematic Review. J Med Syst. 2016;40(3):1–13. PMID: 26748792

21. Schoeppe S, Alley S, Van Lippevelde W, Bray NA, Williams SL, Duncan MJ, et al. Efficacy of interventions that use apps to improve diet, physical activity and sedentary behaviour: A systematic review. Int J Behav Nutr Phys Act. International Journal of Behavioral Nutrition and Physical Activity; 2016;13(1). PMID: 27927218

22. Jee H. Review of researches on smartphone applications for physical activity promotion in healthy adults. J Exerc Rehabil. 2017;13(1):3–11. PMID: 28349027

23. Stuckey MI, Carter SW, Knight E. The role of smartphones in encouraging physical activity in adults. Int J Gen Med. 2017;10:293–303. PMID: 28979157

24. Sullivan AN, Lachman ME. Behavior Change with Fitness Technology in Sedentary Adults: A Review of the Evidence for Increasing Physical Activity. Front Public Heal. 2017;4(January). PMID: 10968301

25. Duncan M, Murawski B, Short CE, Rebar AL, Schoeppe S, Alley S, et al. Activity Trackers Implement Different Behavior Change Techniques for Activity, Sleep, and Sedentary Behaviors. Interact J Med Res. 2017;6(2):e13. PMID: 28807889

26. Mercer K, Li M, Giangregorio L, Burns C, Grindrod K. Behavior Change Techniques Present in Wearable Activity Trackers: A Critical Analysis. JMIR mHealth uHealth. 2016;4(2):e40. PMID: 27122452

27. Lyons EJ, Lewis ZH, Mayrsohn BG, Rowland JL. Behavior change techniques implemented in electronic lifestyle activity monitors: A systematic content analysis. J Med Internet Res. 2014;16(8):e192. PMID: 25131661

28. Straiton N, Alharbi M, Bauman A, Neubeck L, Gullick J, Bhindi R, et al. The validity and reliability of consumer-grade activity trackers in older, community-dwelling adults: A systematic review. Maturitas. Elsevier; 2018;112(March):85–93. PMID: 29704922

29. de Vries HJ, Kooiman TJM, van Ittersum MW, van Brussel M, de Groot M. Do activity monitors increase physical activity in adults with overweight or obesity? A systematic review and meta-analysis. Obesity. 2016;24(10):2078–2091. PMID: 27670401

30. Ridgers ND, McNarry MA, Mackintosh KA. Feasibility and Effectiveness of Using Wearable Activity Trackers in Youth: A Systematic Review. JMIR mHealth uHealth. 2016;4(4):e129. PMID: 27881359

31. Lewis ZH, Lyons EJ, Jarvis JM, Baillargeon J. Using an electronic activity monitor system as an intervention modality: A systematic review Health behavior, health promotion and society. BMC Public Health. BMC Public Health; 2015;15(1). PMID: 26104189

32. Evenson KR, Goto MM, Furberg RD. Systematic review of the validity and reliability of consumer-wearable activity trackers. Int J Behav Nutr Phys Act. International Journal of Behavioral Nutrition and Physical Activity; 2015;12(1). PMID: 26684758

33. Van Remoortel H, Louvaris Z, Vogiatzis I, Langer D, Gimeno-Santos E, Glendenning A, et al. Validity of activity monitors in health and chronic disease: a systematic review. Int J Behav Nutr Phys Act. 2012;9(1):84. PMID: 22776399

34. Michie S, Abraham C, Whittington C, McAteer J, Gupta S. Effective Techniques in Healthy Eating and Physical Activity Interventions: A Meta-Regression. Heal Psychol. 2009;28(6):690–701. PMID: 19916637

35. Williams SL, French DP. What are the most effective intervention techniques for changing physical activity self-efficacy and physical activity behaviour - And are they the same? Health Educ Res. 2011;26(2):308–322. PMID: 21321008

36. Olander EK, Fletcher H, Williams S, Atkinson L, Turner A, French DP. What are the most effective techniques in changing obese individuals’ physical activity self-efficacy and behaviour: A systematic review and meta-analysis. Int J Behav Nutr Phys Act. 2013;10:1–15. PMID: 23452345

37. Brannon EE, Cushing CC. A systematic review: is there an app for that? Translational science of pediatric behavior change for physical activity and dietary interventions. J Pediatr Psychol. 2015;40(4):373–384. PMID: 25502745

38. O’Brien N, McDonald S, Araújo-Soares V, Lara J, Errington L, Godfrey A, et al. The features of interventions associated with long-term effectiveness of physical activity interventions in adults aged 55–70 years: a systematic review and meta-analysis. Health Psychol Rev. 2015;9(4):417–433. PMID: 25689096

39. Samdal GB, Eide GE, Barth T, Williams G, Meland E. Effective behaviour change techniques for physical activity and healthy eating in overweight and obese adults; systematic review and meta-regression analyses. Int J Behav Nutr Phys Act. International Journal of Behavioral Nutrition and Physical Activity; 2017;14(1):1–14. PMID: 28351367

40. Toscos T, Faber A, Connelly K, Upoma AM. Encouraging physical activity in teens can technology help reduce barriers to physical activity in adolescent girls? Proc 2nd Int Conf Pervasive Comput Technol Healthc 2008, PervasiveHealth. 2008;3(Group 3):218–221.

41. Watterson T. Changes in Attitudes and Behaviors toward Physical Activity, Nutrition, and Social Support for Middle School Students Using the AFIT App as a Supplement to Instruction in a Physical Education Class. ProQuest LLC. 2012;(January):1–166.

42. Lu F, Turner K, Murphy B. Reducing adolescent obesity with a mobile fitness application: Study results of youth age 15 to 17. 2013 IEEE 15th Int Conf e-Health Networking, Appl Serv Heal 2013. 2013;(Healthcom):554–558.

43. Van Dantzig S, Geleijnse G, Van Halteren AT. Toward a persuasive mobile application to reduce sedentary behavior. Pers Ubiquitous Comput. 2013;17(6):1237–1246. PMID: 89894772

44. Glynn LG, Hayes PS, Casey M, Glynn F, Alvarez-Iglesias A, Newell J, et al. SMART MOVE - a smartphone-based intervention to promote physical activity in primary care: study protocol for a randomized controlledGlynn, L. G., Hayes, P. S., Casey, M., Glynn, F., Alvarez-Iglesias, A., Newell, J., … Murphy, A. W. (2013). SMART MOVE - a. Trials. 2013;14(1):157. PMID: 23714362

45. Glynn LG, Hayes PS, Casey M, Glynn F, Alvarez-Iglesias A, Newell J, et al. Effectiveness of a smartphone application to promote physical activity in primary care: The SMART MOVE randomised controlled trial. Br J Gen Pract. 2014;64(624):384–391. PMID: 24982490

46. Bond DS, Thomas JG, Raynor HA, Moon J, Sieling J, Trautvetter J, et al. B-MOBILE - A smartphone-based intervention to reduce sedentary time in overweight/obese individuals: A within-subjects experimental trial. PLoS One. 2014;9(6). PMID: 24964010

47. Duncan M, Vandelanotte C, Kolt GS, Rosenkranz RR, Caperchione CM, George ES, et al. Effectiveness of a web- and mobile phone-based intervention to promote physical activity and healthy eating in middle-Aged males: Randomized controlled trial of the manup study. J Med Internet Res. 2014;16(6). PMID: 24927299

48. Lu F, Lemonde M. Reducing adolescent obesity with a social networking mobile fitness application. 2014 IEEE 16th Int Conf e-Health Networking, Appl Serv Heal 2014. 2014;429–434.

49. Smith JJ, Morgan PJ, Plotnikoff RC, Dally KA, Salmon J, Okely AD, et al. Rationale and study protocol for the “Active Teen Leaders Avoiding Screen-time” (ATLAS) group randomized controlled trial: An obesity prevention intervention for adolescent boys from schools in low-income communities. Contemp Clin Trials. Elsevier Inc.; 2014;37(1):106–119. PMID: 24291151

50. Smith JJ, Morgan PJ, Plotnikoff RC, Dally KA, Salmon J, Okely AD, et al. Smart-Phone Obesity Prevention Trial for Adolescent Boys in Low-Income Communities: The ATLAS RCT. Pediatrics. 2014;134(3):e723–e731. PMID: 25157000

51. Recio-Rodríguez JI, Martín-Cantera C, González-Viejo N, Gómez-Arranz A, Arietaleanizbeascoa MS, Schmolling-Guinovart Y, et al. Effectiveness of a smartphone application for improving healthy lifestyles, a randomized clinical trial (EVIDENT II): Study protocol. BMC Public Health. 2014;14(1):1–13. PMID: 24628961

52. Recio-Rodriguez JI, Agudo-Conde C, Martin-Cantera C, González-Viejo M, Fernandez-Alonso MC, Arietaleanizbeaskoa MS, et al. Short-term effectiveness of a mobile phone app for increasing physical activity and adherence to the mediterranean diet in primary care: A randomized controlled trial (EVIDENT II study). J Med Internet Res. 2016;18(12):1–13. PMID: 27993759

53. Blackman KCA, Zoellner J, Kadir A, Dockery B, Johnson SB, Almeida FA, et al. Examining the Feasibility of Smartphone Game Applications for Physical Activity Promotion in Middle School Students. Games Health J. 2015;4(5):409–419. PMID: 26287931

54. Direito A, Jiang Y, Whittaker R, Maddison R. Smartphone apps to improve fitness and increase physical activity among young people: Protocol of the Apps for IMproving FITness (AIMFIT) randomized controlled trial. BMC Public Health. BMC Public Health; 2015;15(1):1–12. PMID: 26159834

55. Direito A, Jiang Y, Whittaker R, Maddison R. Apps for IMproving FITness and increasing physical activity among young people: The AIMFIT pragmatic randomized controlled trial. J Med Internet Res. 2015;17(8):1–13. PMID: 26316499

56. Garde A, Umedaly A, Abulnaga SM, Robertson L, Junker A, Chanoine JP, et al. Assessment of a Mobile Game (“MobileKids Monster Manor”) to Promote Physical Activity Among Children. Games Health J. 2015;4(2):149–158. PMID: 26181809

57. Martin SS, Feldman DI, Blumenthal RS, Jones SR, Post WS, McKibben RA, et al. mActive: A randomized clinical trial of an automated mHealth intervention for physical activity promotion. J Am Heart Assoc. 2015;4(11):1–9. PMID: 26553211

58. Rabbi M, Pfammatter A, Zhang M, Spring B, Choudhury T. Automated Personalized Feedback for Physical Activity and Dietary Behavior Change With Mobile Phones: A Randomized Controlled Trial on Adults. JMIR mHealth uHealth. 2015;3(2):e42. PMID: 25977197

59. Pellegrini CA, Steglitz J, Johnston W, Warnick J, Adams T, McFadden HG, et al. Design and protocol of a randomized multiple behavior change trial: Make Better Choices 2 (MBC2). Contemp Clin Trials. Elsevier Inc.; 2015;41:85–92. PMID: 25625810

60. Spring B, Pellegrini C, McFadden HG, Pfammatter AF, Stump TK, Siddique J, et al. Multicomponent mHealth intervention for large, sustained change in multiple diet and activity risk behaviors: The make better choices 2 randomized controlled trial. J Med Internet Res. 2018;20(6):1–13. PMID: 29921561

61. Choi JW, Lee J hyeon, Vittinghoff E, Fukuoka Y. mHealth Physical Activity Intervention: A Randomized Pilot Study in Physically Inactive Pregnant Women. Matern Child Health J. 2016;20(5):1091–1101. PMID: 26649879

62. Duncan MJ, Vandelanotte C, Trost SG, Rebar AL, Rogers N, Burton NW, et al. Balanced: A randomised trial examining the efficacy of two self-monitoring methods for an app-based multi-behaviour intervention to improve physical activity, sitting and sleep in adults. BMC Public Health. BMC Public Health; 2016;16(1):1–14. PMID: 27473327

63. Harries T, Eslambolchilar P, Rettie R, Stride C, Walton S, Van Woerden HC. Effectiveness of a smartphone app in increasing physical activity amongst male adults: A randomised controlled trial. BMC Public Health. BMC Public Health; 2016;16(1):1–10. PMID: 27590255

64. King AC, Hekler EB, Grieco LA, Winter SJ, Sheats JL, Buman MP, et al. Effects of three motivationally targeted mobile device applications on initial physical activity and sedentary behavior change in midlife and older adults: A randomized trial. PLoS One. 2016;11(6):1–16. PMID: 27352250

65. Lubans DR, Smith JJ, Peralta LR, Plotnikoff RC, Okely AD, Salmon J, et al. A school-based intervention incorporating smartphone technology to improve health-related fitness among adolescents: Rationale and study protocol for the NEAT and ATLAS 2.0 cluster randomised controlled trial and dissemination study. BMJ Open. 2016;6(6):368–373. PMID: 24149423

66. Lubans DR, Smith JJ, Plotnikoff RC, Dally KA, Okely AD, Salmon J, et al. Assessing the sustained impact of a school-based obesity prevention program for adolescent boys: The ATLAS cluster randomized controlled trial. Int J Behav Nutr Phys Act. International Journal of Behavioral Nutrition and Physical Activity; 2016;13(1):149–152. PMID: 17388708

67. Rospo G, Valsecchi V, Bonomi AG, Thomassen IW, van Dantzig S, La Torre A, et al. Cardiorespiratory Improvements Achieved by American College of Sports Medicine’s Exercise Prescription Implemented on a Mobile App. JMIR mHealth uHealth. 2016;4(2):e77. PMID: 27339153

68. Voth EC, Oelke ND, Jung ME. A Theory-Based Exercise App to Enhance Exercise Adherence: A Pilot Study. JMIR mHealth uHealth. 2016;4(2):e62. PMID: 27307134

69. Walsh JC, Corbett T, Hogan M, Duggan J, McNamara A. An mHealth Intervention Using a Smartphone App to Increase Walking Behavior in Young Adults: A Pilot Study. JMIR mHealth uHealth. 2016;4(3):e109. PMID: 27658677

70. Shin DW, Joh HK, Yun JM, Kwon HT, Lee H, Min H, et al. Design and baseline characteristics of participants in the Enhancing Physical Activity and Reducing Obesity through Smartcare and Financial Incentives (EPAROSFI): A pilot randomized controlled trial. Contemp Clin Trials. Elsevier B.V.; 2016;47:115–122. PMID: 26744232

71. Shin DW, Yun JM, Shin JH, Kwon H, Min HY, Joh HK, et al. Enhancing physical activity and reducing obesity through smartcare and financial incentives: A pilot randomized trial. Obesity. 2017;25(2):302–310. PMID: 28063226

72. Fanning J, Roberts S, Hillman CH, Mullen SP, Ritterband L, McAuley E. A smartphone “app”-delivered randomized factorial trial targeting physical activity in adults. J Behav Med. Springer US; 2017;40(5):712–729. PMID: 28255750

73. Gaudet J, Gallant F, Bélanger M. A Bit of Fit: Minimalist Intervention in Adolescents Based on a Physical Activity Tracker. JMIR mHealth uHealth. 2017;5(7):e92. PMID: 28684384

74. Klasnja P, Smith S, Seewald NJ, Lee A, Hall K, Luers B, et al. Efficacy of Contextually Tailored Suggestions for Physical Activity: A Micro-randomized Optimization Trial of HeartSteps. Ann Behav Med. 2018;1–10. PMID: 30192907

75. Korinek E V., Phatak SS, Martin CA, Freigoun MT, Rivera DE, Adams MA, et al. Adaptive step goals and rewards: a longitudinal growth model of daily steps for a smartphone-based walking intervention. J Behav Med. Springer US; 2018;41(1):74–86. PMID: 28918547

76. Recio-Rodriguez JI, Gómez-Marcos MA, Agudo-Conde C, Ramirez I, Gonzalez-Viejo N, Gomez-Arranz A, et al. EVIDENT 3 Study: a randomized, controlled clinical trial to reduce inactivity and caloric intake in sedentary and overweight or obese people using a smartphone application: study protocol. Medicine (Baltimore). 2018;97(2):e9633. PMID: 29480874

77. Van Woudenberg TJ, Bevelander KE, Burk WJ, Smit CR, Buijs L, Buijzen M. A randomized controlled trial testing a social network intervention to promote physical activity among adolescents. BMC Public Health. BMC Public Health; 2018;18(1):1–11. PMID: 29685112

78. Rabin C, Bock B. Desired Features of Smartphone Applications Promoting Physical Activity. Telemed e-Health. 2011;17(10):801–803. PMID: 22010977

79. Dennison L, Morrison L, Conway G, Yardley L. Opportunities and challenges for smartphone applications in supporting health behavior change: Qualitative study. J Med Internet Res. 2013;15(4):475–478. PMID: 19942754

80. Ehlers DK, Huberty JL. Middle-Aged Women’s Preferred Theory-Based Features in Mobile Physical Activity Applications. J Phys Act Heal. 2014;11(7):1379–1385. PMID: 24368818

81. Gowin M, Cheney M, Gwin S, Franklin Wann T. Health and Fitness App Use in College Students: A Qualitative Study. Am J Heal Educ. 2014;46(4):223–230.

82. Miyamoto SW, Henderson S, Young HM, Pande A, Han JJ. Tracking Health Data Is Not Enough: A Qualitative Exploration of the Role of Healthcare Partnerships and mHealth Technology to Promote Physical Activity and to Sustain Behavior Change. JMIR mHealth uHealth. 2016;4(1):e5. PMID: 26792225

83. Arteaga SM, Kudeki M, Woodworth A, Kurniawan S. Mobile system to motivate teenagers’ physical activity. Proc 9th Int Conf Interact Des Child - IDC ’10. 2010;1. PMID: 17381235

84. Ayubi SU, Parmanto B. PersonA: Persuasive social network for physical Activity. Proc Annu Int Conf IEEE Eng Med Biol Soc EMBS. 2012;2153–2157. PMID: 23366348

85. Hebden L, Cook A, Van Der Ploeg HP, Allman-Farinelli M. Development of smartphone applications for nutrition and physical activity behavior change. J Med Internet Res. 2012;14(4):1–12. PMID: 23611892

86. King AC, Hekler EB, Grieco LA, Winter SJ, Sheats JL, Buman MP, et al. Harnessing Different Motivational Frames via Mobile Phones to Promote Daily Physical Activity and Reduce Sedentary Behavior in Aging Adults. PLoS One. 2013;8(4):245–260. PMID: 23638127

87. Lubans DR, Smith JJ, Skinner G, Morgan PJ. Development and Implementation of a Smartphone Application to Promote Physical Activity and Reduce Screen-Time in Adolescent Boys. Front Public Heal. 2014;2(May):1–11. PMID: 24904909

88. Alnasser A, Sathiaseelan A, Al-Khalifa A, Marais D. Development of ‘Twazon’: An Arabic App for Weight Loss. JMIR Res Protoc. 2016;5(2):e76. PMID: 27185568

89. Vos S, Janssen M, Goudsmit J, Lauwerijssen C, Brombacher A. From Problem to Solution: Developing a Personalized Smartphone Application for Recreational Runners following a Three-step Design Approach. Procedia Eng. Elsevier B.V.; 2016;147(6):799–805. PMID: 29657368

90. Middelweerd A, te Velde SJ, Mollee JS, Klein MC, Brug J. App-Based Intervention Combining Evidence-Based Behavior Change Techniques With a Model-Based Reasoning System to Promote Physical Activity Among Young Adults (Active2Gether): Descriptive Study of the Development and Content. JMIR Res Protoc. 2018;7(12):e185. PMID: 30578198

91. Chang T-R, Kaasinen E, Kaipainen K. What influences users’ decisions to take apps into use? Proc 11th Int Conf Mob Ubiquitous Multimed - MUM ’12. 2012;1.

92. Middelweerd A, van der Laan DM, van Stralen MM, Mollee JS, Stuij M, te Velde SJ, et al. What features do Dutch university students prefer in a smartphone application for promotion of physical activity? A qualitative approach. Int J Behav Nutr Phys Act. 2015;12(1):1–11. PMID: 25889577

93. Arteaga SM, González VM, Kurniawan S, Benavides RA. Mobile games and design requirements to increase teenagers’ physical activity. Pervasive Mob Comput. Elsevier B.V.; 2012;8(6):900–908.

94. Arteaga SM, Kurniawan S. Designing an application to motivate teenagers’ physical activity. IEEE Int Conf Commun. 2012;6106–6110. PMID: 19425278

95. Lu F, Turner K. Improving adolescent fitness attitudes with a mobile fitness game to combat obesity in youth. IEEE Consum Electron Soc Int Games Innov Conf IGIC. 2013;148–151.

96. Casey M, Hayes PS, Glynn F, Ólaighin G, Heaney D, Murphy AW, et al. Patients’ experiences of using a smartphone application to increase physical activity: The SMART MOVE qualitative study in primary care. Br J Gen Pract. 2014;64(625):500–508. PMID: 25071063

97. Herrmann LK, Kim J. The fitness of apps: a theory-based examination of mobile fitness app usage over 5 months. mHealth. 2017;3(November 2016):2–2. PMID: 28293619

98. Abraham C, Michie S. A Taxonomy of Behavior Change Techniques Used in Interventions. Heal Psychol. 2008;27(3):379–387. PMID: 18624603

99. Wiehe SE, Carroll AE, Liu GC, Haberkorn KL, Hoch SC, Wilson JS, et al. Using GPS-enabled cell phones to track the travel patterns of adolescents. Int J Health Geogr. 2008;7:1–11. PMID: 18495025

100. Wiehe SE, Hoch SC, Liu GC, Carroll AE, Wilson JS, Fortenberry JD. Adolescent Travel Patterns: Pilot Data Indicating Distance from Home Varies by Time of Day and Day of Week. J Adolesc Heal. 2008;42(4):418–420. PMID: 18346668

101. Kukafka R, Khan SA, Kaufman D, Mark J. An evidence-based decision aid to help patients set priorities for selecting among multiple health behaviors. AMIA . Annu Symp proceedings AMIA Symp. American Medical Informatics Association; 2009;2009:343–347. PMID: 20351877

102. Lu H, Yang J, Liu Z, Lane ND, Choudhury T, Campbell AT. The Jigsaw continuous sensing engine for mobile phone applications. Proc 8th ACM Conf Embed Networked Sens Syst - SenSys ’10. 2010;71. PMID: 22107704

103. Michie S, Ashford S, Sniehotta FF, Dombrowski SU, Bishop A, French DP. A refined taxonomy of behaviour change techniques to help people change their physical activity and healthy eating behaviours: The CALO-RE taxonomy. Psychol Heal. 2011;26(11):1479–1498. PMID: 21678185

104. Michie S, van Stralen MM, West R. The behaviour change wheel: a new method for characterising and designing behaviour change interventions. Implement Sci. BioMed Central; 2011;6:42. PMID: 21513547

105. Riley WT, Rivera DE, Atienza AA, Nilsen W, Allison SM, Mermelstein R. Health behavior models in the age of mobile interventions: Are our theories up to the task? Transl Behav Med. 2011;1(1):53–71. PMID: 21796270

106. Klasnja P, Pratt W. Healthcare in the pocket: Mapping the space of mobile-phone health interventions. J Biomed Inform. Elsevier Inc.; 2012;45(1):184–198. PMID: 21925288

107. Li I, Dey AK, Forlizzi J. Using context to reveal factors that affect physical activity. ACM Trans Comput Interact. 2012;19(1):1–21. PMID: 17511749

108. Nilsen W, Kumar S, Shar A, Varoquiers C, Wiley T, Riley WT, et al. Advancing the Science of mHealth. J Health Commun. Taylor & Francis; 2012;17(sup1):5–10. PMID: 22548593

109. Michie S, Richardson M, Johnston M, Abraham C, Francis J, Hardeman W, et al. The behavior change technique taxonomy (v1) of 93 hierarchically clustered techniques: Building an international consensus for the reporting of behavior change interventions. Ann Behav Med. 2013;46(1):81–95. PMID: 23512568

110. Spring B, Gotsis M, Paiva A, Sprujit-Metz D. Healthy Apps: Mobile Devices for Continuous Monitoring and Intervention. IEEE Pulse. 2013;4(November):34–40. PMID: 24233190

111. Turner-McGrievy GM, Beets MW, Moore JB, Kaczynski AT, Barr-Anderson DJ, Tate DF. Comparison of traditional versus mobile app self-monitoring of physical activity and dietary intake among overweight adults participating in an mHealth weight loss program. J Am Med Informatics Assoc. 2013;20(3):513–518. PMID: 23429637

112. Kumar S, Nilsen WJ, Abernethy A, Atienza A, Patrick K, Pavel M, et al. Mobile Helath Technology evaluation. Am J Prev Med. 2013;45(2):228–236. PMID: 23867031

113. Hirsch JA, James P, Robinson JRM, Eastman KM, Conley KD, Evenson KR, et al. Using MapMyFitness to Place Physical Activity into Neighborhood Context. Front Public Heal. 2014;2(March):1–9. PMID: 24653982

114. Dusseldorp E, van Buuren S, van Genugten L, van Empelen P, Verheijden MW. Combinations of techniques that effectively change health behavior: Evidence from Meta-CART analysis. Heal Psychol. 2013;33(12):1530–1540. PMID: 24274802

115. Case MA, Burwick HA, Volpp KG, Patel MS. Accuracy of smartphone applications and wearable devices for tracking physical activity data. JAMA - J Am Med Assoc. 2015;313(6):625–626. PMID: 25668268

116. Belmon LS, Middelweerd A, te Velde SJ, Brug J. Dutch Young Adults Ratings of Behavior Change Techniques Applied in Mobile Phone Apps to Promote Physical Activity: A Cross-Sectional Survey. JMIR mHealth uHealth. 2015;3(4):e103. PMID: 26563744

117. Kok G, Gottlieb NH, Peters GJY, Mullen PD, Parcel GS, Ruiter RAC, et al. A taxonomy of behaviour change methods: an Intervention Mapping approach. Health Psychol Rev. Taylor & Francis; 2015;10(3):297–312. PMID: 26262912

118. Michie S, Wood CE, Johnston M, Abraham C, Francis JJ, Hardeman W. Behaviour change techniques: The development and evaluation of a taxonomic method for reporting and describing behaviour change interventions (a suite of five studies involving consensus methods, randomised controlled trials and analysis of qualitative da. Health Technol Assess (Rockv). 2015;19(99):1–187. PMID: 26616119

119. Hardcastle SJ, Hancox J, Hattar A, Maxwell-Smith C, Thøgersen-Ntoumani C, Hagger MS. Motivating the unmotivated: how can health behavior be changed in those unwilling to change? Front Psychol. 2015;6(June):1–4. PMID: 26136716

120. Benson AC, Bruce L, Gordon BA. Reliability and validity of a GPS-enabled iPhone^TM^ “app” to measure physical activity. J Sports Sci. 2015;33(14):1421–1428. PMID: 25555093

121. Neubeck L, Lowres N, Benjamin EJ, Freedman S Ben, Coorey G, Redfern J. The mobile revolution-using smartphone apps to prevent cardiovascular disease. Nat Rev Cardiol. Nature Publishing Group; 2015;12(6):350–360. PMID: 25801714

122. Aitken M, Lyle J. Patient adoption of mHealth: use, evidence and remaining barriers to mainstream acceptance. Parsippany, NJ. 2015.

123. Dallinga JM, Mennes M, Alpay L, Bijwaard H, Baart De La Faille-Deutekom M. App use, physical activity and healthy lifestyle: A cross sectional study. BMC Public Health. BMC Public Health; 2015;15(1):1–9. PMID: 26316060

124. Gillman AS, Bryan AD. Effects of Performance Versus Game-Based Mobile Applications on Response to Exercise. Ann Behav Med. 2016;50(1):157–162. PMID: 26362539

125. Stoyanov SR, Hides L, Kavanagh DJ, Zelenko O, Tjondronegoro D, Mani M. Mobile App Rating Scale: A New Tool for Assessing the Quality of Health Mobile Apps. JMIR mHealth uHealth. 2015;3(1):e27. PMID: 25760773

126. Vonholtz LAH, Hypolite KA, Carr BG, Shofer FS, Winston FK, Hanson CW, et al. Use of mobile apps: A patient-centered approach. Acad Emerg Med. 2015;22(6):765–768. PMID: 25998446

127. Thompson D, Peacock O, Western M, Batterham AM. Multidimensional physical activity: An opportunity, not a problem. Exerc Sport Sci Rev. 2015;43(2):67–74. PMID: 25607280

128. Kukafka R, Jeong IC, Finkelstein J. Optimizing Decision Support for Tailored Health Behavior Change Applications. Stud Health Technol Inform. 2015;216:108–112. PMID: 26262020

129. Michie S, Carey RN, Johnston M, Rothman AJ, de Bruin M, Kelly MP, et al. From Theory-Inspired to Theory-Based Interventions: A Protocol for Developing and Testing a Methodology for Linking Behaviour Change Techniques to Theoretical Mechanisms of Action. Ann Behav Med. Annals of Behavioral Medicine; 2016;1–12. PMID: 27401001

130. Edwards EA, Lumsden J, Rivas C, Steed L, Edwards LA, Thiyagarajan A, et al. Gamification for health promotion: systematic review of behaviour change techniques in smartphone apps. BMJ Open. 2016;6(10):e012447. PMID: 27707829

131. Harari GM, Lane ND, Wang R, Crosier BS, Campbell AT, Gosling SD. Using Smartphones to Collect Behavioral Data in Psychological Science: Opportunities, Practical Considerations, and Challenges. Perspect Psychol Sci. 2016;11(6):838–854. PMID: 27899727

132. Lobelo F, Kelli HM, Tejedor SC, Pratt M, McConnell M V., Martin SS, et al. The Wild Wild West: A Framework to Integrate mHealth Software Applications and Wearables to Support Physical Activity Assessment, Counseling and Interventions for Cardiovascular Disease Risk Reduction. Prog Cardiovasc Dis. 2016;58(6):584–594. PMID: 26923067

133. Recio GM, García-Hernández L, Molina Luque R, Salas-Morera L. The role of interdisciplinary research team in the impact of health apps in health and computer science publications: A systematic review. Biomed Eng Online. BioMed Central; 2016;15(1):53–97. PMID: 27454164

134. Stoyanov SR, Hides L, Kavanagh DJ, Wilson H. Development and Validation of the User Version of the Mobile Application Rating Scale (uMARS). JMIR mHealth uHealth. JMIR Publications; 2016;4(2):e72–e72. PMID: 27287964

135. Pham Q, Wiljer D, Cafazzo JA. Beyond the Randomized Controlled Trial: A Review of Alternatives in mHealth Clinical Trial Methods. JMIR mHealth uHealth. 2016;4(3):e107. PMID: 27613084

136. White BK, Burns SK, Giglia RC, Scott JA. Designing evaluation plans for health promotion mHealth interventions: A case study of the Milk Man mobile app. Heal Promot J Aust. 2016;27(3):198–203. PMID: 27784506

137. Lyons EJ, Swartz MC. Motivational Dynamics of Wearable Activity Monitors. Acsms Heal Fit J. 2017;21(5):21–26.

138. Wright SP, Hall Brown TS, Collier SR, Sandberg K. How consumer physical activity monitors could transform human physiology research. Am J Physiol - Regul Integr Comp Physiol. 2017;312(3):R358–R367. PMID: 28052867

139. Choe EK, Abdullah S, Rabbi M, Thomaz E, Epstein DA, Cordeiro F, et al. Semi-Automated Tracking: A Balanced Approach for Self-Monitoring Applications. IEEE Pervasive Comput. 2017;16(1):74–84.

140. Michie S, Yardley L, West R, Patrick K, Greaves F. Developing and evaluating digital interventions to promote behavior change in health and health care: Recommendations resulting from an international workshop. J Med Internet Res. 2017;19(6). PMID: 28663162

141. Carroll JK, Moorhead A, Bond R, LeBlanc WG, Petrella RJ, Fiscella K. Who uses mobile phone health apps and does use matter? A secondary data analytics approach. J Med Internet Res. 2017;19(4):1–9. PMID: 28428170

142. Ernsting C, Dombrowski SU, Oedekoven M, O’Sullivan JL, Kanzler E, Kuhlmey A, et al. Using smartphones and health apps to change and manage health behaviors: A population-based survey. J Med Internet Res. 2017;19(4):1–12. PMID: 28381394

143. Hoj TH, Covey EL, Jones AC, Haines AC, Hall PC, Crookston BT, et al. How Do Apps Work? An Analysis of Physical Activity App Users’ Perceptions of Behavior Change Mechanisms. JMIR mHealth uHealth. 2017;5(8):e114. PMID: 28778846

144. Pirolli P, Mohan S, Venkatakrishnan A, Nelson L, Silva M, Springer A. Implementation Intention and Reminder Effects on Behavior Change in a Mobile Health System: A Predictive Cognitive Model. J Med Internet Res. 2017;19(11):e397. PMID: 29191800

145. Moller AC, Merchant G, Conroy DE, West R, Hekler E, Kugler KC, et al. Applying and advancing behavior change theories and techniques in the context of a digital health revolution: proposals for more effectively realizing untapped potential. J Behav Med. 2017;40(1):85–98. PMID: 28058516

146. Schembre SM, Liao Y, Robertson MC, Dunton GF, Kerr J, Haffey ME, et al. Just-in-time feedback in diet and physical activity interventions: Systematic review and practical design framework. J Med Internet Res. 2018;20(3):1–14. PMID: 29567638

147. Feldman DI, Theodore Robison W, Pacor JM, Caddell LC, Feldman EB, Deitz RL, et al. Harnessing mHealth technologies to increase physical activity and prevent cardiovascular disease. Clin Cardiol. 2018;41(7):985–991. PMID: 29671879

148. McCallum C, Rooksby J, Cindy MG. Evaluating the impact of physical activity apps and wearables: Interdisciplinary review. J Med Internet Res. 2018;20(3):1–20. PMID: 29572200
